# Exploiting a rodent cell block for intrinsic resistance to HIV-1 gene expression in human T cells

**DOI:** 10.1128/mbio.00420-23

**Published:** 2023-09-07

**Authors:** Ryan T. Behrens, Jyothi Krishnaswamy Rajashekar, James W. Bruce, Edward L. Evans, Amelia M. Hansen, Natalia Salazar-Quiroz, Lacy M. Simons, Paul Ahlquist, Judd F. Hultquist, Priti Kumar, Nathan M. Sherer

**Affiliations:** 1 McArdle Laboratory for Cancer Research and Carbone Cancer Center, University of Wisconsin-Madison, Madison, Wisconsin, USA; 2 Institute for Molecular Virology, University of Wisconsin-Madison, Madison, Wisconsin, USA; 3 Department of Internal Medicine, Section of Infectious Diseases, Yale University School of Medicine, New Haven, Connecticut, USA; 4 Morgridge Institute for Research, University of Wisconsin-Madison, Madison, Wisconsin, USA; 5 Division of Infectious Diseases, Northwestern University Feinberg School of Medicine, Chicago, Illinois, USA; Rutgers-Robert Wood Johnson Medical School, Piscataway, New Jersey, USA; National Cancer Institute, Frederick, Maryland, USA

**Keywords:** human immunodeficiency virus, lentiviruses, transcription, nuclear export, Cyclin T1, CCNT1, Tat, CRM1, XPO1, Rev, genome engineering, CRISPR/Cas9

## Abstract

HIV-1 virion production is inefficient in cells derived from mice and other rodents reflecting cell-intrinsic defects to interactions between the HIV-1 auxiliary proteins Tat and Rev and host dependency factors CCNT1 (Cyclin T1) and XPO1 (exportin-1, also known as CRM1), respectively. In human cells, Tat binds CCNT1 to enhance viral RNA transcription and Rev recruits XPO1 to mediate the nuclear export of intron-containing viral RNA. In mouse cells, Tat’s interactions with CCNT1 are inefficient, mapped to a single species-specific residue Y261 instead of C261 in humans. Rev interacts poorly with murine XPO1, mapped to a trio of amino acids T411/V412/S414 instead of P411/M412/F414 in humans. To determine if these discrete species-specific regions of otherwise conserved housekeeping proteins represent viable targets for inhibiting HIV-1 replication in humans, herein, we employed CRISPR/Cas9 to recode the relevant regions of CCNT1 and XPO1 in human CD4+ T cells. While efforts to modify XPO1 were inconclusive, we generated isogenic CCNT1.C261Y cell lines exhibiting remarkable resistance to HIV-1 Tat, exhibiting near total inactivation of viral gene expression for all X4- and R5-tropic HIV-1 strains tested, as well as the more distantly related primate lentiviruses HIV-2 and SIV_agm_. Induction of viral reactivation using latency reversal agents (LRAs) was also restricted in CCNT1.C261Y cells. These studies validate a minor and naturally occurring, species-specific difference in a conserved human host factor as a compelling potential target for achieving broad-acting cell-intrinsic resistance to HIV’s post-integration phases.

**Importance:**

Unlike humans, mice are unable to support HIV-1 infection. This is due, in part, to a constellation of defined minor, species-specific differences in conserved host proteins needed for viral gene expression. Here, we used precision CRISPR/Cas9 gene editing to engineer a “mousified” version of one such host protein, cyclin T1 (CCNT1), in human T cells. CCNT1 is essential for efficient HIV-1 transcription, making it an intriguing target for gene-based inactivation of virus replication. We show that isogenic cell lines engineered to encode CCNT1 bearing a single mouse-informed amino acid change (tyrosine in place of cysteine at position 261) exhibit potent, durable, and broad-spectrum resistance to HIV-1 and other pathogenic lentiviruses, and with no discernible impact on host cell biology. These results provide proof of concept for targeting *CCNT1* in the context of one or more functional HIV-1 cure strategies.

## INTRODUCTION

Cell-intrinsic barriers to human immunodeficiency type 1 (HIV-1) replication can confer life-long resistance to HIV-associated diseases in humans. A hallmark example is a naturally occurring 32 bp deletion in the *CCR5* gene (*CCR5Δ32*) that reduces cell surface levels of CCR5, the surface receptor for transmitted “R5-tropic” forms of HIV-1 (1 – 3). Allogeneic bone marrow transplant using cells homozygous for *CCR5Δ32* has contributed to the only known instances of HIV/AIDS functional cure (4 – 7). These cases, known as the Berlin, London, and New York patients, demonstrated that drug-free remission or cure is possible through targeted inactivation of CCR5. However, the potential drawbacks to gene-targeted CCR5-based cures include the potential for viral evolution of altered receptor tropism (8) (e.g*.,* outgrowth of CXCR4-tropic HIV-1) and the ∆32 allele being linked to increased risk of symptomatic flavivirus infections (9 – 11). These issues underscore the need to consider additional gene targets in the context of cell-based functional cures.

In this context, studies of HIV-1 replication in non-human mammals have revealed a trove of species-specific gene barriers to infection (12, 13). For example, rhesus macaques resist HIV-1 due to the activities of endogenous APOBEC3G and TRIM5 proteins that disrupt intact viral genome delivery to targeted cells (14, 15). Other forms of HIV-1 resistance can be attributed to species-specific differences to so-called host-dependency factors (HDFs); cellular proteins hijacked by the virus to carry out specific life cycle stages. For example, mice and other rodents lack HIV-compatible versions of both CCR5 and the primary attachment receptor CD4, thereby resisting viral entry. Exogenous expression of human CD4 and CCR5 was shown to alleviate viral entry blocks in some rodent cell lines (16 – 28). However, these provisions were insufficient to restore virus replication due to additional rodent cell blocks affecting HIV-1’s post-integration stages (29 – 50).

Of the post-integration rodent cell blocks, those affecting the essential HIV-1 accessory proteins Tat and Rev are best characterized. In human cells, HIV-1 Tat drives viral transcription by recruiting the positive transcription elongation factor b (p-TEFb) complex, consisting of a heterodimer of the CDK9 kinase and its regulatory partner CCNT1, to the *trans*-activation response element (TAR) structure present in the 5′ portion of initiated viral transcripts (51). CDK9 phosphorylates the C-terminal domain of RNA polymerase II to promote processivity and synthesis of full-length viral RNAs (51). Human CCNT1 encodes a cysteine at residue 261 (C261) thought to coordinate a zinc ion that stabilizes the functional Tat/p-TEFb/TAR complex (34 – 36). The murine ortholog of CCNT1 encodes a tyrosine at the equivalent position (Y261) and is associated with poor Tat-dependent transcription (34 – 36).

By contrast, in human cells, HIV-1 Rev enables the nuclear export of unspliced and partially spliced viral RNAs that would otherwise remain retained in the nucleus (51). In the nucleus, Rev binds to and multimerizes on the Rev response element (RRE) structure present on unspliced and partially spliced transcripts and engages the XPO1 (exportin-1, also called CRM1) nuclear export machinery (51). Human XPO1 features a species-specific surface element defined by a proline, methionine, and phenylalanine at residues 411, 412, and 414, respectively (P411, M412, and F414 in humans and T411, V412, and S414 in mice), that we and others have shown is needed for efficient Rev activity (48 – 50). This “patch-like” element is likely important for stabilizing the formation of functional Rev/RRE/XPO1 export complexes consisting of multiple XPO1 proteins (52, 53). An alternative but nonmutually exclusive model suggests that Rev/RRE complexes bind with greater affinity to human XPO1 compared to the murine counterpart (54).

Based on this knowledge, in this study, we asked if these discrete, naturally occurring, rodent-specific HDF blocks to HIV-1 Tat and Rev function could be engineered into human CD4+ Jurkat T cells, taking advantage of precision CRISPR/Cas9 genome editing. We provide strong evidence that human CCNT1 can be “mousified,” i.e., modified to engender a rodent cell-like block to Tat function, and as proof-of-concept in the context of an antiviral target, isolated isogenic CCNT1.C261Y cell lines that were effectively resistant to HIV-1 Tat activity and viral gene expression despite the C261Y edit having no discernible effects on CCNT1-regulated host cell functions. Furthermore, we demonstrate that CCNT1.C261Y cells are likely to exhibit cell-intrinsic resistance to all known HIV-1 strains and subtypes, inducing a state of forced viral latency consistent with a “block-and-lock” provirus inactivation scenario. By contrast, we were unable to validate effects on Rev function attributable to the species-specific trio of XPO1 residues. Collectively, these results highlight the potential value of a species-specific domain of human host factor CCNT1 as a target for anti-HIV intervention.

## RESULTS

### A dual-reporter strategy to screen for cell-intrinsic blocks to early- (Tat-driven) and late-stage (Rev-driven) HIV-1 gene expression

**Fig 1:**
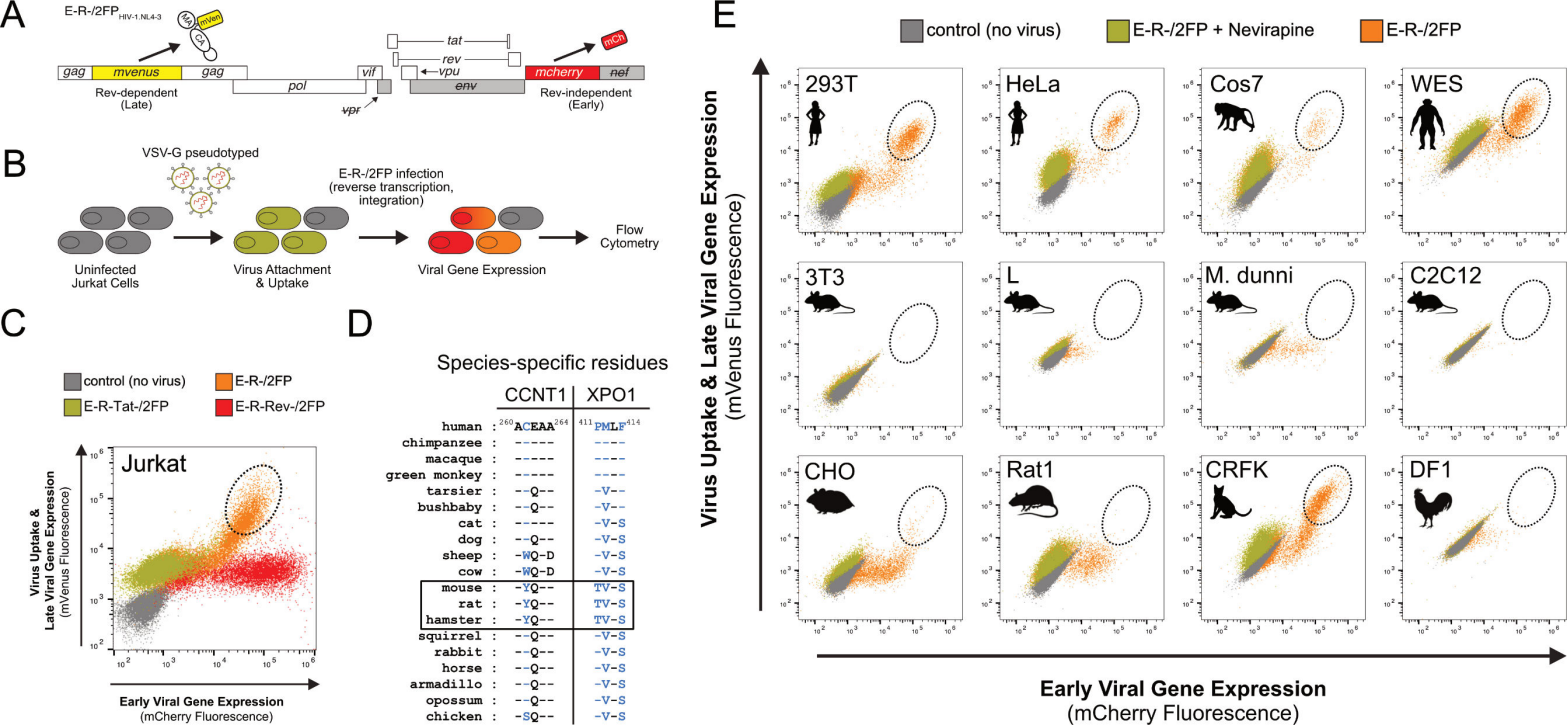
Development of a dual-reporter assay to screen for blocks to early-stage (Tat-driven) and late-stage (Rev-driven) HIV-1 gene expression. (**A**) Viral genome diagram of the two-color, HIV-1.NL4-3-derived viral reporter construct, E-R-/2FP. Early, Rev-independent viral gene expression is reported by mCherry, encoded within the *nef* locus. Late, Rev-dependent viral gene expression is reported by mVenus, encoded in *gag* as an in-frame insertion between the matrix (MA) and capsid (CA) protein-coding regions. Gray rectangles denote inactivated *env*, *vpr*, and *nef* genes to limit virus replication to a single cycle. (**B**) E-R-/2FP reporter assay schematic. VSV-G pseudotyped virus particles containing E-R-/2FP genomes are delivered to target cells. Prior to infection, target cells (gray) can become mVenus-positive due to virion-associated MA-mVenus during virus particle uptake (gold). Following proviral insertion, early (red) and late (orange) viral gene expression is reported by *de novo* mCherry and mVenus expression, respectively. (**C**) Fluorescence profiles of Jurkat E6-1 cells transduced with wild type (WT), Tat- or Rev-deficient E-R-/2FP, or untransduced control cells. Cells exhibiting robust early and late viral gene expression are indicated (dashed circle). Dot plot is an overlay of the four indicated cell populations. (**D**) Species-specific CCNT1 or XPO1 residues that govern HIV-1 Tat and Rev activity, respectively (blue). CCNT1 residue 261 governs Tat activity. XPO1 residues 411, 412, and 414 govern Rev activity. Rodents predicted from species-specific residue identity to exhibit poor Tat and Rev activities are indicated (boxed). (**E**) E-R-/2FP screen of various mammalian cell lines. Dashed circles highlight cells exhibiting robust early and late viral gene expression. Nevirapine treatment to block E-R-/2FP infection was to facilitate the identification of productively infected cell populations.

So that we could screen for cell-intrinsic blocks to Tat- and Rev-dependent HIV-1 gene expression, we first devised a flow cytometric assay to capture fluorescence profiles of cell populations infected with a two-color HIV-1 reporter virus (E-R-/2FP, Fig. 1A); E-R-/2FP encodes *mCherry* from the *nef* locus to monitor early, Tat-dependent but Rev-independent gene expression and *mVenus* as a Gag fusion (matrix [MA]-mVenus-capsid [CA]) reporting on late, both Tat- and Rev-dependent gene expression. Inactivating mutations in *env* and *vpr* genes (E-/R-) limited virus replication to a single round and reduced cytopathic effects (Fig. 1B).

To validate our reporter, we infected Jurkat T cells with our E-R-/2FP virus or control versions engineered to carry inactivating mutations in *tat* (E-R-Tat-/2FP) or *rev* (E-R-Rev-/2FP) (Fig. 1C). Compared to mock-infected cells (gray dots), E-R-/2FP-infected cells (orange dots) yielded a robust population of bright double-positive cells (orange dots in dashed oval), consistent with efficient early and late viral gene expression phases. E-R-Rev-/2FP-infected cells (red dots) were mCherry-bright but mVenus-dim, consistent with the activity of Tat, but not Rev. As expected, E-R-Tat-/2FP-infected cells (gold dots) lacked both mCherry and mVenus fluorescence but, like the E-R-Rev/2FP cells, were moderately mVenus-positive due to cellular uptake of the Gag-mVenus fluorescent virus particles. In sum, E-R-/2FP control variants confirmed our dual reporter as capable of quantifying the relative activities of Tat and Rev with single-cell resolution.

For further validation, we next tested our E-R-/2FP assay using a panel of informative cell lines predicted to encode permissive or non-permissive species-specific determinants encoded by *CCNT1* and *XPO1*, respectively (Fig. 1D, shown in blue). Expectedly, primate cell lines including 293T, HeLa, Cos7, and WES exhibited fluorescence profiles similar to infected Jurkat T cells, confirming robust Tat and Rev activity (Fig. 1E). In contrast, mouse cell lines including 3T3, L, and C2C12 exhibited deficiencies to Tat- and Rev-dependent gene expression, yielding few to no late stage, bright double-positive cells. Interestingly, M. dunni, Rat1 and Chinese hamster ovary (CHO) cells were largely deficient in late gene expression but exhibited a moderate degree of early, Rev-independent gene expression, illustrating that the transcription block was not absolute in these cell types despite their encoding CCNT1 Y261. Infected DF1 chicken cells, that encode a serine at the equivalent position of CCNT1 C261, were also only modestly permissive for early viral gene expression. CRFK cat cells were interesting in that, despite encoding a XPO1 patch region containing rodent-like V412 and S414 residues, these cells exhibited strong Rev activity, potentially highlighting the relative importance of the XPO1 P411 residue or other yet to be mapped determinants. For all lines, treatment with the reverse transcriptase inhibitor nevirapine (gold dots) served as a control to demarcate productively infected populations (orange dots) from uninfected cells (gray dots) (Fig. 1E). Taken together, we concluded that our E-R-/2FP assay faithfully detected cell-intrinsic differences to Tat and Rev activities and would, therefore, be useful to screen modified human T cell populations for equivalent defects.

### Host-targeted CRISPR/Cas9 knock-in strategy to abrogate HIV-1 gene expression in human T cells

**Fig 2:**
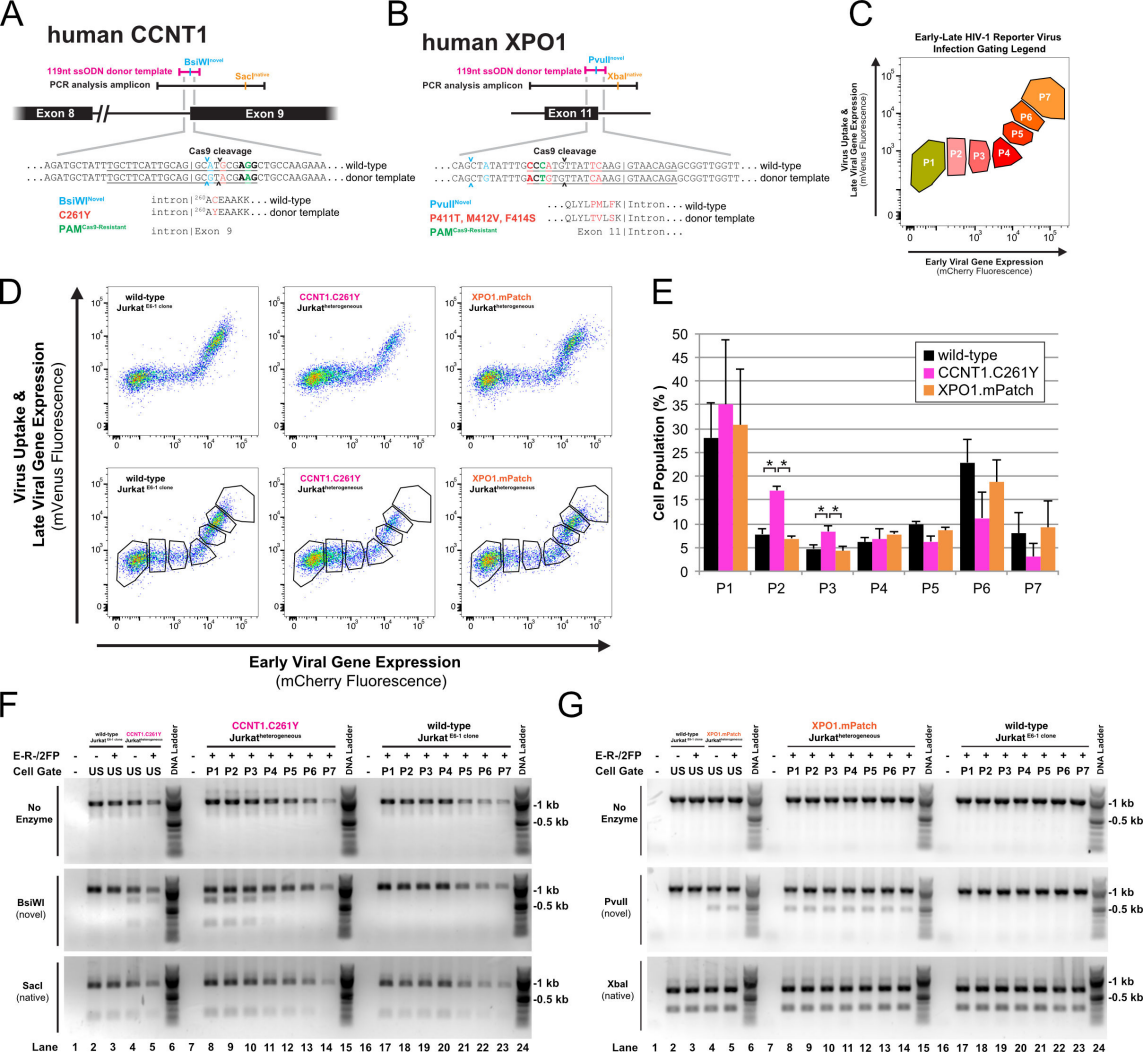
CRISPR/Cas9 knock-in strategy to abrogate Tat or Rev function in human T cells. (**A**) Design schematic for *CCNT1* gene knock-in editing. Approximate sequence lengths of the donor template (pink) and PCR amplicon (black) were used to analyze the genomic locus. DNA sequences relevant to the edit design are shown; the PAM (bold) and guide RNA target (underlined) specify the Cas9 cleavage site (arrows). Mutations engineered into the donor template are color-coded according to their intended use. CCNT1 protein-coding sequences are shown with substitutions color-coded to match the relevant DNA mutation. (**B**) Design schematic for *XPO1* gene knock-in editing labeled as in Fig. 2A. (**C**) FACS gating legend for sorted populations P1–P7. Gates P1–P4 contain cells exhibiting early, Tat-dependent viral gene expression. Gates P5–P7 contain cells exhibiting late, Tat-/Rev-dependent viral gene expression. (**D**) Fluorescence profiles of *CCNT1- or XPO1-*edited or control cells infected with E-R-/2FP with (bottom row) or without (top row) P1-P7 gate overlays. P411T, M412V, and F414S substitutions collectively termed “mPatch.” (**E**) Percentage of cells inoculated with E-R-/2FP within gates P1-P7 for the *CCNT1*- (pink), *XPO1*- (orange)*,* and control-edited (black) cell populations. Error bars represent the standard deviation from the mean for three independent experiments. A two-tailed Student’s *t*-test with Welch’s correction was used to compare the indicated populations (*P ≤ 0.05). (**F**) Sensitivity of PCR amplicons from E-R-/2FP-inoculated, *CCNT1*-edited or control cells before and after FACS. PCR amplicons (top row) were digested with BsiWI (middle row) or SacI (bottom row) to screen for amplicons containing gene knock-in for each sorted population (P1–P7, lanes 8–14 and 17–23). Unsorted E-R-/2FP-inoculated or no infection control cell populations (US, lanes 1–5) were analyzed to demonstrate input amplicon sensitivities to the indicated restriction enzymes. (**G**) Sensitivity of PCR amplicons from E-R-/2FP-inoculated *XPO1*-edited or control cells before and after FACS. PCR amplicons (top row) were digested with PvuII (middle row) or XbaI (bottom row) to screen for amplicons containing gene knock-in for each sorted population (P1–P7, lanes 8–14 and 17–23). Unsorted E-R-/2FP-inoculated or no infection control cell populations (US, lanes 1–5) were analyzed to demonstrate input amplicon sensitivities to the indicated restriction enzymes.

Next, we designed CRISPR/Cas9 gene knock-in schemes to determine if we could engineer rodent-informed Tat (CCNT1)- or Rev (XPO1)-dependent HIV-1 gene expression blocks into human CD4+ T cells, using the Jurkat E6-1 cell line as a tractable model system. Gene knock-in modifications were performed by co-delivering single-stranded oligodeoxynucleotide (ssODN) donor templates along with recombinant Cas9 in complex with guide RNAs bearing complementary sequences to targeted loci. For human *CCNT1*, the endogenous “TGC” codon for cysteine at residue position 261 was replaced with “TAC” in the ssODN, directing a substitution for the mouse-encoded tyrosine (Fig. 2A, C261Y, modifications in red). Codon mutations were similarly made for the combined proline, methionine, and phenylalanine residues at positions 411, 412, and 414 encoded by human *XPO1*, with the goal of replacing them with the mouse-encoded threonine, valine, and serine, respectively (Fig. 2B, P411T, M412V, and F414S, modifications in red; collectively termed, “mPatch”). Silent mutations in ssODNs encoding novel restriction enzyme sites (BsiWI for *CCNT1* and PvuII for *XPO1*) allowed for knock-in detection using restriction enzyme digest (Fig. 2A and B, modifications in blue). Protospacer adjacent motifs (PAMs) were also mutated to prevent Cas9-mediated cleavage of introduced template sequences (Fig. 2A and B, modifications in green).

After editing, Jurkat cell populations were screened using the E-R-/2FP assay described for Fig. 1. We hypothesized that, if refractory, *CCNT1*- or *XPO1*-modified cells would exhibit profiles similar to those observed for rodent cell lines (see Fig. 1E). To better resolve changes, we further augmented the assay to include fluorescence-associated cell sorting (FACS) using seven cell gates (Fig. 2C) allowing us to separate cell populations to represent the progression of viral gene expression beginning with mCherry-positive, Tat-dependent but Rev-independent stages (P1–P4) and ending with dual positive, late-stage Rev-dependent stages (P5–P7). Comparing *CCNT1*-targeted cells to both wild type (WT) and *XPO1*-edited cells, we observed marked increases to the number of cells in P2 and P3 gates, consistent with blocks to Tat function in a significant subset of *CCNT1*-edited cells (Fig. 2E). By contrast, the number of cells in any gate of the *XPO1*-targeted cell population was not significantly altered (Fig. 2E).

Because spontaneous insertion or deletion events can manifest off-target effects, we also analyzed each sorted pool to confirm allele knock-in frequency using restriction digests (P1–P7). Because the *CCNT1* knock-in was predicted to manifest a Tat block, we hypothesized that alleles bearing the *CCNT1* knock-in signature would be enriched in cells within gates P1–P4 compared to those in the P5–P7 gates, confirmed by BsiWI sensitivity in the heterogeneous amplicon pools (Fig. 2F). Analysis of *XPO1* knock-in by PvuII digestion sensitivity confirmed modifications; however, we observed no allele enrichment in any single gated population (Fig. 2G). Overall, these results suggested that a mouse-encoded CCNT1 HIV-1 block to Tat function could be engineered into a human T cell population with relatively high efficiency; however, that modifying XPO1 to achieve a Rev block was less feasible.

### *CCNT1.C261*Y Jurkat T cells suppress HIV-1 Tat activity

**Fig 3:**
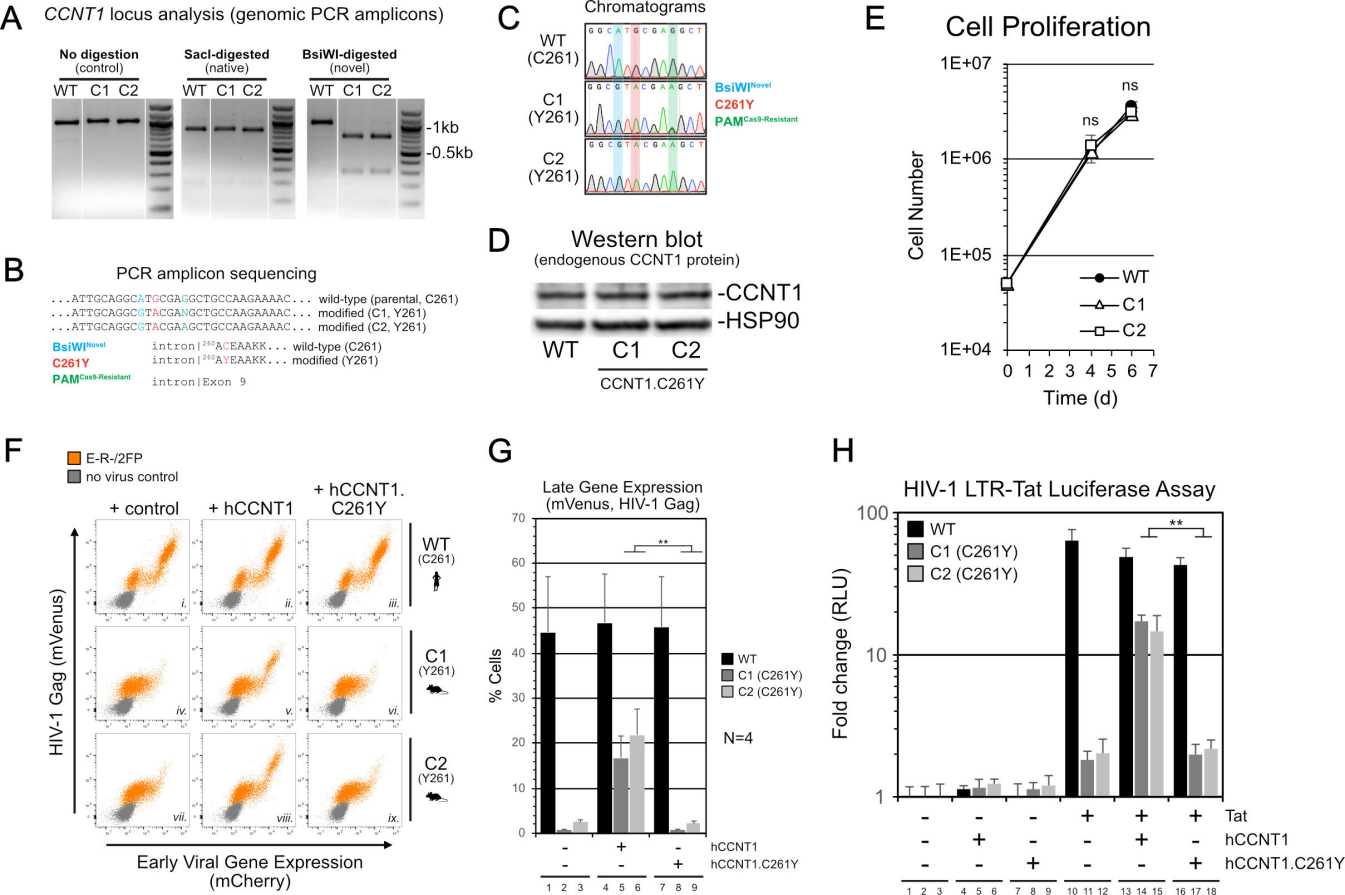
Homozygous CCNT1.C261Y Jurkat T cells potently suppress HIV-1 Tat activity. (**A**) Analysis of the *CCNT1* locus by PCR amplicon restriction digest for CCNT1.C261Y clones, C1 and C2, compared to WT parental cells. (**B**) Sequence analysis summary for CCNT1.C261Y cells. (**C**) Sequence read chromatograms for CCNT1.C261Y cells. Read peaks of loci targeted for mutation are color-coded by intended purpose. (**D**) Western blot analysis of CCNT1 protein levels in CCNT1.C261Y cells. (**E**) Cell proliferation analysis of CCNT1.C261Y cells. A two-tailed Student’s *t*-test with Welch’s correction was used to compare C1 or C2 to wild-type parental cells at the specified time point (ns, not significant). (**F**) Exogenous hCCNT1, but not hCCNT1.C261Y, restored the E-R-/2FP gene expression and the wild-type fluorescence profile in CCNT1.C261Y clones, C1 and C2. Vectors encoding wild-type hCCNT1 (middle column) or hCCNT1.C261Y (right column) or control (left column) were delivered to the parental Jurkat cell line (top row, panels i–iii) or CCNT1.C261Y clones C1 (middle row, panels iv–vi) and C2 (bottom row, panels vii–ix) then infected with E-R-/2FP virus (orange) and analyzed by flow cytometry. (**G**) Quantitative summary of late gene expression was presented in Fig. 3F. A two-tailed Student’s *t*-test with Welch’s correction was used to compare CCNT1.C261Y cells expressing exogenous hCCNT1 to those expressing hCCNT1.C261Y. (**H**) Exogenous hCCNT1, but not hCCNT1.C261Y, restores Tat activity in an infection-independent Tat/LTR-luciferase reporter assay. Plasmids encoding luciferase under control of the HIV-1 LTR and HIV-1 Tat were delivered in combination with either hCCNT1, hCCNT1.C261Y, or a vector control by transfection. A two-tailed Student’s *t*-test with Welch’s correction was used to compare CCNT1.C261Y cells expressing exogenous hCCNT1 to those expressing hCCNT1.C261Y.

Data indicating strong and stable blocks to Tat function in *CCNT1*-edited Jurkat cells prompted us to isolate two *CCNT1.C261Y* cell lines, named C1 and C2, encoding *CCNT1* loci confirmed to be entirely sensitive to BsiWI digestion (Fig. 3A) and seamlessly edited, evidenced by the absence of unintended mutations at the targeted locus (Fig. 3B and C). CCNT1.C261Y protein levels were equivalent to non-edited CCNT1 in the parental cell line (Fig. 3D), and we observed no effects on cell proliferation (Fig. 3E). The E-R-/2FP reporter assay confirmed that the modified cells exhibited a fluorescence profile consistent with loss of Tat function (Fig. 3F, compare panels i–iv and vii) and, importantly, significantly rescued by exogenous expression of human CCNT1 but not a CCNT1.C261Y mutant transgene, delivered using retroviral vectors (Fig. 3F and G).

To address Tat activity independently of infection, we next employed a transient reporter assay wherein a firefly luciferase gene under transcriptional control of the HIV-1 LTR (LTR-Luc) was expressed in the presence or absence of Tat in WT and both C261Y cell lines (Fig. 3H) (55). When Tat was expressed in the presence of the LTR-Luc construct, we observed a luciferase signal >30-fold higher in the parental cell line relative to either C261Y clone (Fig. 3H, compare lane 10–11 and 12) confirming severe but not absolute disruption to Tat-driven gene expression, and again significantly rescued when co-expressed with WT human CCNT1 but not the CCNT1.C261Y mutant (Fig. 3H, compare lane 14–17; compare lane 15–18). Together, these results demonstrated that otherwise permissive human T cells can be rendered resistant to Tat function engendered by a single amino acid substitution at position 261.

### The CCNT1.C261Y modification potently inhibits diverse HIV-1 strains as well as HIV-2 and SIV_agm_

**Fig 4:**
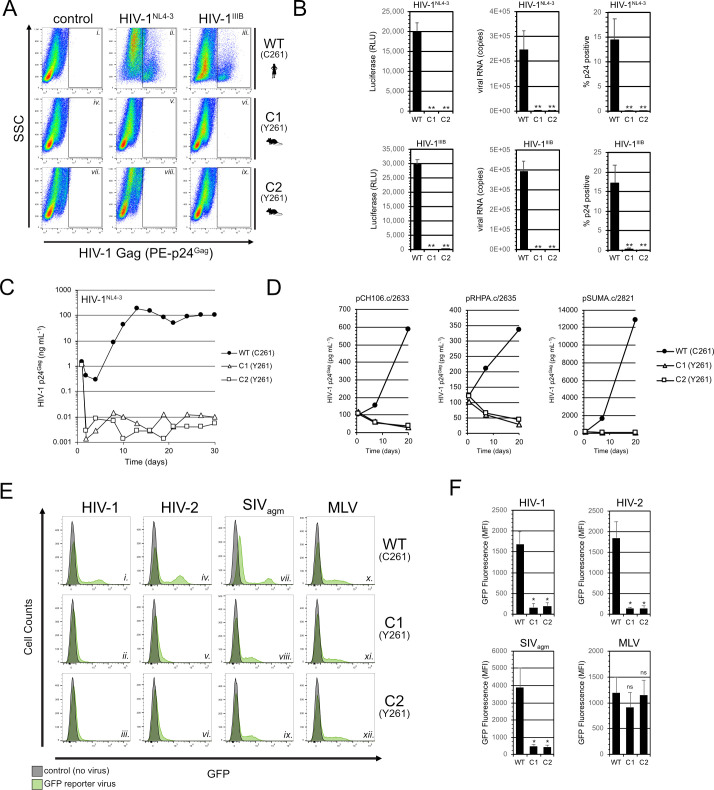
The CCNT1.C261Y modification potently blocks the replication of diverse HIV-1 strains and also inhibits HIV-2 and SIV_agm_ gene expression. (**A**) CCNT1.C261Y cells are resistant to HIV-1 replication. Representative p24^Gag^ antigen staining of HIV-1-infected cells 10 days post infection. (**B**) Quantitative analysis of HIV-1.NL4-3- and HIV-1.IIIB-infected cell cultures 10 days post infection by supernatant infectivity (TZM-bl luciferase assays, left bar graphs) and viral RNA content (qRT-PCR, middle bar graphs), as well as infected cell populations (p24^Gag^ staining, right bar graphs). A two-tailed Student’s *t*-test with Welch’s correction was used to compare C1 or C2 measurements to wild type (**P ≤ 0.01). (**C**) Cell cultures inoculated with HIV-1.NL4-3 were sampled periodically over 1 month span for p24^Gag^ antigen measurements. (**D**) Cell cultures inoculated with the CCR5-tropic, transmitted, or founder (T/F) clones, HIV-1.CH106, HIV-1.RHPA, or HIV-1.SUMA were sampled periodically over 3-week span for p24^Gag^ antigen measurements. (**E**) CCNT1.C261Y cells limit viral gene expression for HIV-1 (panels i–iii), HIV-2 (panels iv–vi), and SIV_agm_ (panels vii–ix) but not MLV (panels x–xii). Flow cytometry histograms from one representative experiment are shown. (**F**) Mean fluorescence intensities (MFIs) from transduced, GFP-positive cell populations were measured in three independent experiments. A two-tailed Student’s *t*-test with Welch’s correction was used to compare C1 or C2 measurements to wild type (*P ≤ 0.05; ns, not significant).

Having confirmed a Tat-specific block using viral reporter assays, we next examined the effects of C261Y on *bona fide* HIV-1 in cell cultures, under conditions of continuous virus replication and spread. We first infected each cell line with either of two CXCR4 (X4)-tropic HIV-1 strains, NL4-3 or IIIB, at a low multiplicities of infection (MOI 0.5) and maintained the cultures for 10 days to allow for multiple rounds of virus replication. After 10 days, compared to parental cells, there was no evidence of infectious virion production in infected CCNT1.C261Y cultures based on measuring levels of viral p24^Gag^ protein and infectivity in the supernatants, correlating with a striking, commensurate lack of viral RNA expression (Fig. 4A, compare panels ii–v and viii and compare panels iii–vi and ix, and Fig. 4B). To test if there was potential for viral escape, we performed infections with a higher viral dose (HIV-1.NL4-3, MOI ~10) and maintained the cultures for 30 days to allow greater time for viral adaptation and outgrowth. No signs of virus amplification following initial infection were evident, confirming a strong and persistent block (Fig. 4C). Because most transmitted strains of HIV-1 are R5-tropic, we also tested transmitted/founder strains HIV-1.CH106.c/2633, HIV-1.RHPA.c/2635, and HIV-1.SUMA.c/2821 in CCNT1 WT and C261Y lines engineered to stably express CCR5 (Fig. 4D) (56). Again, compared to the R5-positive parental cells, we observed no evidence of virus replication in R5-positive CCNT1.C261Y cells monitored over 20 days post infection.

Because primate lentiviruses code for divergent Tat proteins that similarly drive transcription elongation (57), we tested if CCNT1.C261Y cells would also inhibit transcription using validated GFP-encoded viral vector systems derived from HIV-1, HIV-2, and simian immunodeficiency virus from the African green monkey (SIV_agm_) (58, 59) while also including the Tat-independent murine leukemia virus (MLV) as a control. For each vector except for MLV, GFP fluorescence was significantly diminished in the CCNT1.C261Y cell lines compared to the parental cells, consistent with the notion that most if not all primate lentiviruses rely on CCNT1 C261 to achieve efficient transcription (Fig. 4E with quantitative comparisons shown in Fig. 4F). Taken together, the C261Y substitution appeared to be sufficient to achieve remarkably broad-spectrum resistance to primate lentiviral gene expression.

### Infected CCNT1.C261Y cells establish an intrinsic “block-and-lock” provirus inactivation scenario

**Fig 5:**
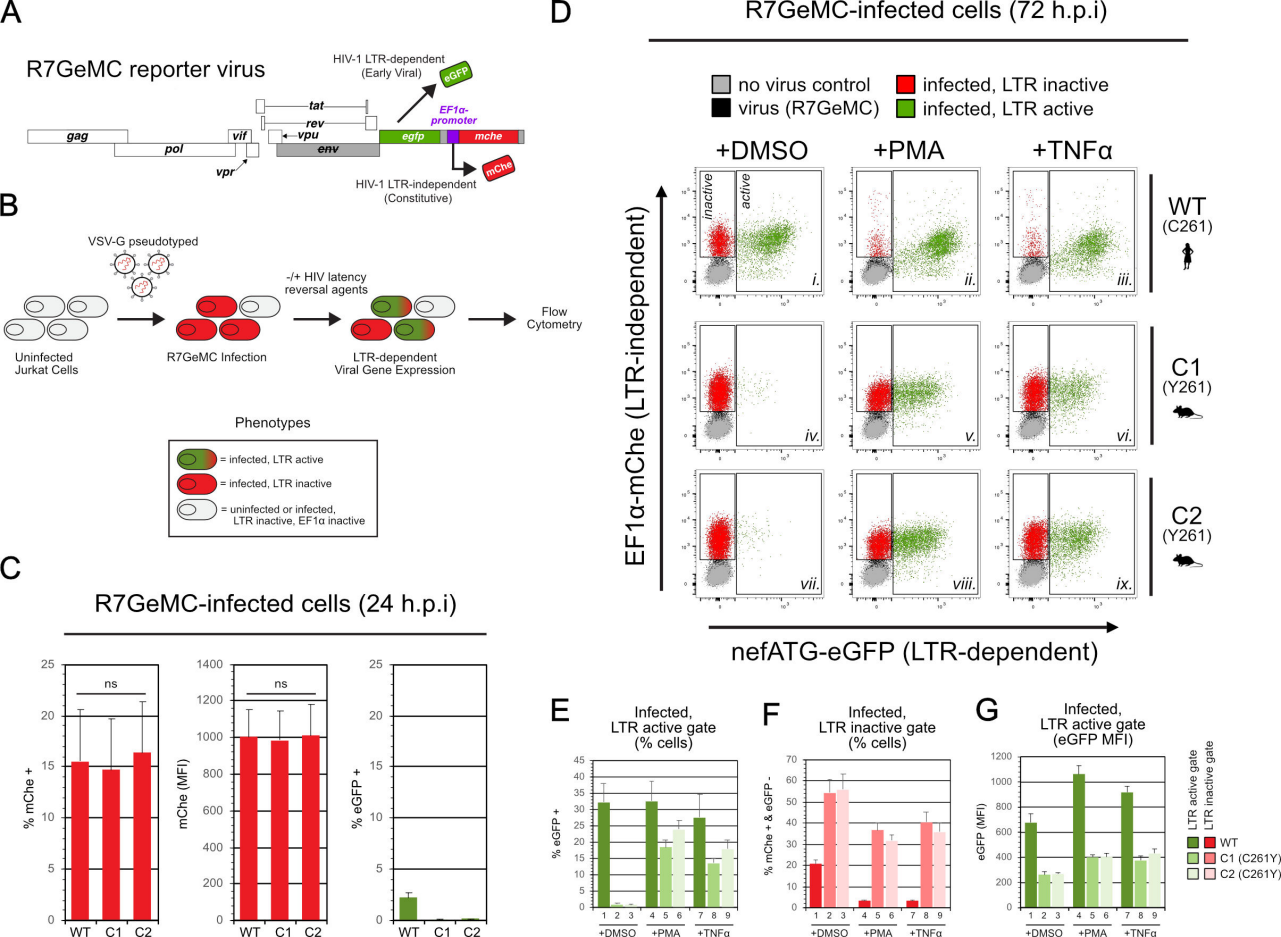
CCNT1.C261Y can be infected by HIV-1 but effectively establish an intrinsic “block-and-lock” provirus inactivation scenario. (**A**) Virus genome diagram of R7GeMC, an established two-color reporter virus (60) designed to discern transcriptionally active or inactive HIV-1 proviruses. LTR-dependent viral gene expression is reported by eGFP. Inactive proviruses are reported by mCherry, encoded downstream of the constitutively active EF1α promoter. Gray rectangles denote inactivated *env* and *nef* genes to limit virus replication to a single cycle. (**B**) R7GeMC reporter assay schematic. Each cell line was inoculated with VSV-G pseudotyped R7GeMC. Twenty-four hours post inoculation, cells were sampled for fluorescence measurements and the remaining were washed to remove input virus. Cells were split into equivalent cultures and treated with HIV-1 latency reversal agents (LRAs). Seventy-two hours post inoculation, cells were harvested for fluorescence measurements. (**C**) Wild-type and CCNT1.C261Y cells are similarly susceptible to R7GeMC. Twenty-four hours post inoculation, eGFP- and mCherry-positive cells and mean fluorescence intensities were measured. A two-tailed Student’s *t*-test with Welch’s correction was used to compare measurements for C1 or C2 to wild type (ns, not significant). (**D**) CCNT1.C261Y cells inhibit integrated R7GeMC proviruses from escaping viral latency, even in the presence of LRAs. Overlaid dot plots of the four indicated cell populations harvested 72 h post inoculation. (**E**) Percentage of eGFP-positive infected cells, indicating cells exhibiting active, LTR-dependent viral gene expression (Fig. 5D, “active” gate percentages). (**F**) Percentage of infected mCherry-positive, but transcriptionally inactive eGFP-negative cells, indicating cells with integrated proviruses that lack LTR-dependent viral gene expression (Fig. 5D, “inactive” gate percentages). (**G**) eGFP mean fluorescence intensity (MFI) in the transcriptionally active infected cell populations (Fig. 5D, eGFP MFI in “active” gates).

Given the canonical role of CCNT1 residue C261 in HIV-1 infection, we hypothesized that CCNT1.C261Y cells would be susceptible to HIV-1 infection but would yield a forced latency phenotype downstream of proviral integration due to Tat inactivation. To test this hypothesis, we infected WT and C261Y cell lines with an established latency-tracking HIV-1 reporter virus, R7GeMC, that encodes two reporter cassettes engineered into the *nef*-coding position: an *egfp* gene responsive to the viral LTR promoter and Tat and a *mcherry* gene under control of a constitutively-active EF1α promoter (Fig. 5A; assay depiction in Fig. 5B) (60). At 24 h post infection, we observed equivalent numbers of mCherry-positive (~15%) cells for both the CCNT1.C261Y and parental control cells, confirming that HIV-1 entry is not altered in CCNT1.C261Y cell lines and that the antiviral effects occur post-integration (Fig. 5C).

A recently described HIV-1 functional cure strategy termed “block-and-lock” aims to use drugs to silence HIV-1 Tat-driven gene expression (“block”) long enough for cell epigenetic modifications to progressively restrict the capacity of HIV-1 to reactivate (“lock”) should drug therapy be discontinued (61). To test the hypothesis that, should CCNT1.C261Y cells become infected, they would effectively represent a cell-intrinsic “block-and-lock” scenario, we tested the responsiveness of R7GeMC-infected WT and C261Y cells to latency reversal agents that act by potentiating basal, Tat-independent viral gene expression. One day following R7GeMC infection, cultures were washed to remove input virus and treated with the LRAs phorbol 12-myristate 13-acetate (PMA; 10 ng mL^−1^), tumor necrosis factor alpha (TNFα; 10 ng mL^−1^), or with DMSO as a vehicle control. In the absence of LRAs, the wild-type cell population featured >28-fold more eGFP-positive cells relative to either infected CCNT1.C261Y cell line, consistent with CCNT1.C261Y cells harboring a significantly greater proportion of inactive proviruses (Fig. 5D, compare “active” gate in panels i–iv and vii; quantification in Fig. 5E; conversely, compare “inactive” gate in panels i–iv and vii; quantification in Fig. 5F). PMA and TNFα treatment increased the proportion of mCherry- and eGFP-positive CCNT1.C261Y cells (compare “active” gate in panels iv–v and vi and panels vii–viii and ix; quantification in Fig. 5E). However, the net viral gene expression was highly attenuated relative to untreated wild-type cells (compare “active” gate in panel ii–v and viii; and iii to vi and ix; quantification in Fig. 5E through G). In sum, the CCNT1.C261Y modification enforced a state of viral latency in human T cells consistent with the inhibition of HIV-1 Tat and severely restricted the potential for viral reactivation in the presence of LRAs, collectively achieving the defining characteristics of a “block-and-lock” scenario even at early time points post-infection and in the absence of suppressive agents.

### Editing CCNT1.C261Y has no discernible effects on p-TEFb activity or major effects on transcription in HIV-1-negative cells

Finally, the capacity of CCNT1.C261Y cells to achieve cell-intrinsic HIV-1 restriction in the absence of apparent effects on cell viability prompted us to investigate the potential for impacts of the CCNT1.C261Y substitution on p-TEFb complex formation or activity in the context of the cellular host (and independent of HIV-1 infection). The p-TEFb complex is conserved in eukaryotes and regulates transcription elongation for a substantial proportion of RNA polymerase II-dependent genes (62 – 64). While an important role for CCNT1 residue 261 in HIV-1 transcription was obvious, any potential effects of targeting C261 on cellular gene expression would need to be considered should this residue/region be considered in the context of an antiviral strategy.

**Fig 6:**
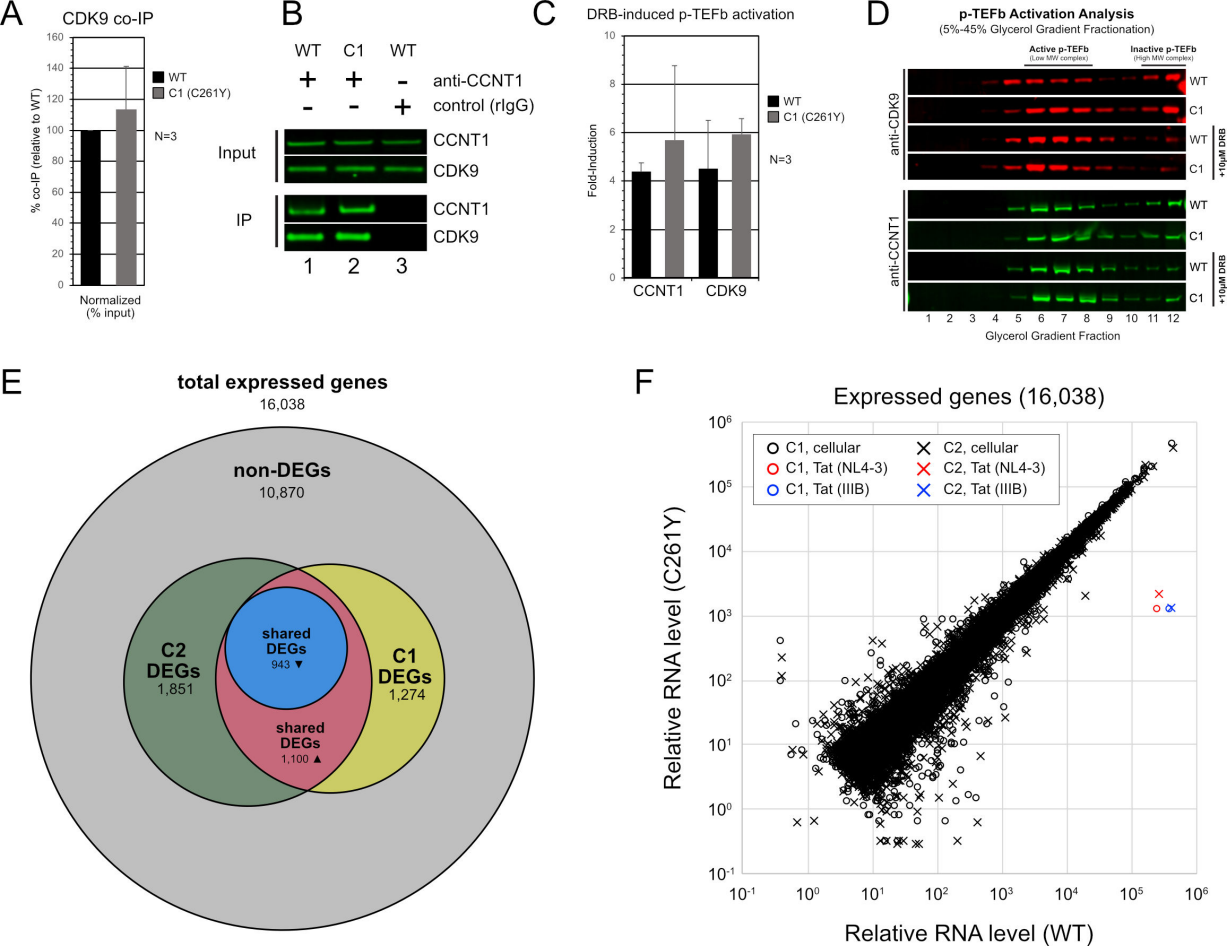
Analysis of p-TEFb and cellular transcriptomes in CCNT1.C261Y cells. (**A**) CCNT1.C261Y does not alter CDK9-CCNT1 interactions. Protein complexes purified from lysates using CCNT1 antisera were analyzed for CDK9 protein levels. Recombinant IgG was used to control for immunoprecipitation (IP) specificity. CDK9 levels in IP complexes were measured by semi-quantitative western blot. Error bars represent the standard deviation from the mean for three independent experiments. (**B**) Representative western blot for data was presented in Fig. 6A. (**C**) CCNT1.C261Y does not change DRB-induced p-TEFb activation responses. Parental and CCNT1.C261Y clone C1 were cultured in the presence or absence of 10 µM DRB and lysed. Cellular lysates were subjected to glycerol gradient fractionation (5%–45%) and the levels of CCNT1 and CDK9 protein were measured by semi-quantitative western blot. Error bars represent the standard deviation from the mean for three independent experiments. (**D**) Representative western blot for data presented in Fig. 6C. (**E**) Venn diagram of the summarized differential gene expression (DGE) analysis comparing CCNT1.C261Y and wild-type transcriptomes (DESeq2). Total expressed genes (16,038) were defined by a baseMean count value of ≥5. Differentially expressed genes (DEGs, 5,168) were defined by an adjusted *P*-value (P*) ≤ 0.05. DEGs unique to C1 (1,274), C2 (1,851), or shared in both (2,043) are defined. DEGs that are up-regulated (1,100) or down-regulated (943) in both C1 and C2 compared to wild-type cells are defined. (**F**) Relative levels of cellular and Tat-dependent RNA in wild-type and C261Y cell lines. Relative RNA abundance (normalized read counts from DESeq2 analysis) for all expressed genes (16,038) in uninfected cells are plotted (C1, circles; C2, crosses). Tat-dependent HIV-1 transcript levels (RT-qPCR copy number; from Fig. 4B) are embedded for context (red, NL4-3; blue, IIIB).

To do so, we first compared CCNT1-CDK9 interactions in WT and CCNT1.C261Y cells using co-immunoprecipitation (co-IP) analysis. Comparable amounts of CDK9 were pulled down by CCNT1 or CCNT1.C261Y from respective cell lysates, indicating that p-TEFb complexes were unperturbed by the CCNT1 C261Y substitution (Fig. 6A, representative blot in Fig. 6B). Previous studies had demonstrated that treatment of cells with an RNA polymerase II inhibitor, 5,6-dichlorozo-1-β-d-ribofuranosylbenzimidazole (DRB), leads to a shift of p-TEFb from a high molecular weight complex to a low molecular weight complex, reflecting the release of p-TEFb from its transcriptionally repressive 7SK RNA scaffold (65, 66). Upon DRB-treatment, we observed an induction of p-TEFb in CCNT1.C261Y cells (i.e., a decrease in the inactive form of p-TEFb and increase in the active form) comparable to that observed in parental cells (Fig. 6C, representative blot in Fig. 6D). Thus, p-TEFb responses to signal-induced stimuli appeared to be intact in CCNT1.C261Y cells.

To employ a more global approach, we next sequenced and compared the transcriptomes of uninfected parental cells to those of either the C1 or C2 CCNT1.C261Y cell clones, performing differential gene expression (DGE) analysis using DESeq2 with a low stringency threshold (adjusted *P*-value, P* ≤ 0.05, baseMean count value of ≥5). This analysis indicated that 12.7% of differentially expressed genes (DEGs) were potentially linked to the C261Y substitution. However, the magnitude of these differences was very low (typically <twofold) compared to the hyperstimulatory effects of C261 on Tat-dependent gene expression (typically >100-fold) observed in our replication experiments (Fig. 6F). These data underscored the relative specificity of CCNT1.C261 in regulating viral versus cellular transcription.

**Fig 7:**
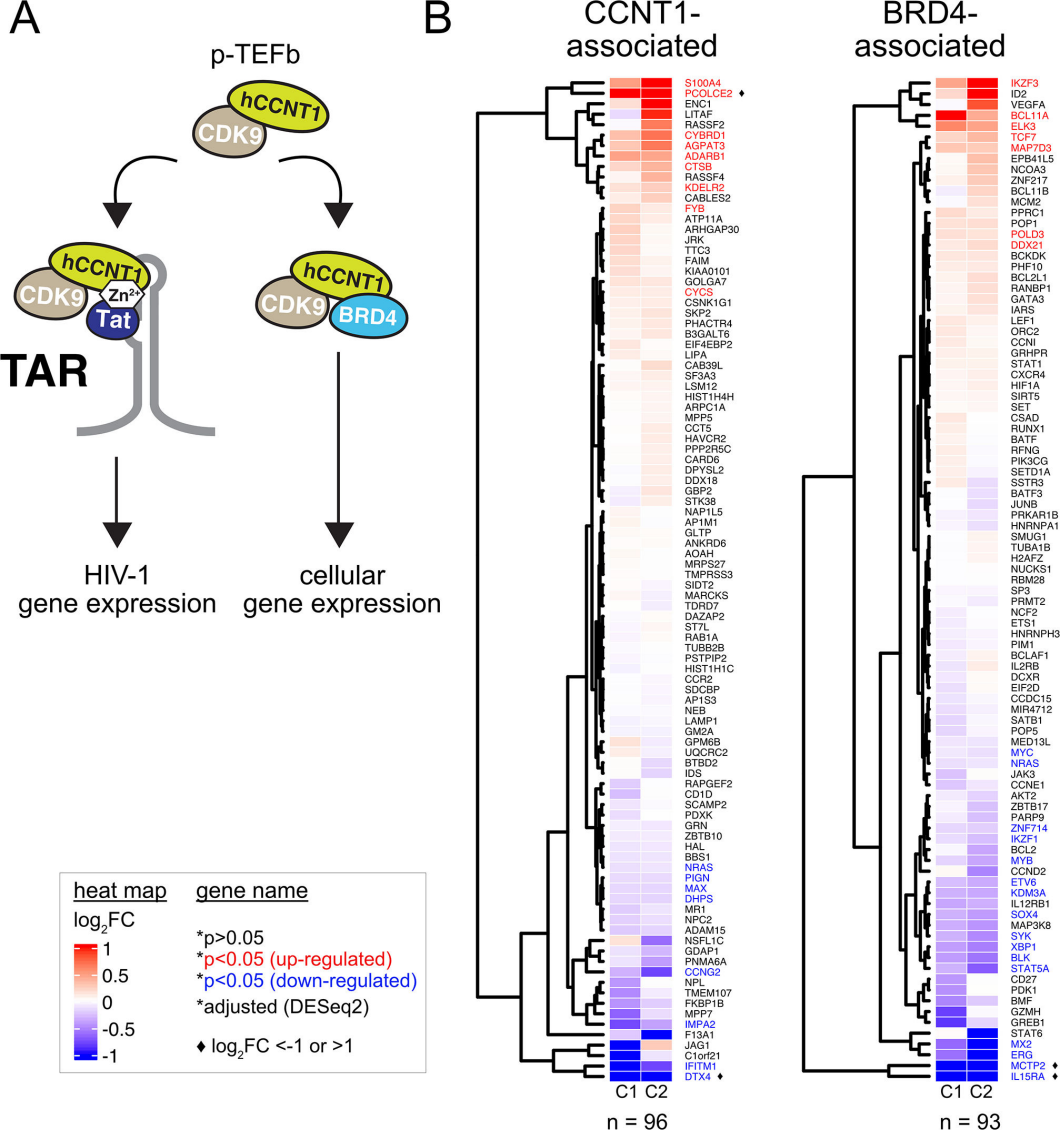
CCNT1- and BRD4-associated genes are largely unperturbed in CCNT1.C261Y cells. (**A**) Cartoon depiction of p-TEFb-dependent gene expression using either BRD4 or Tat/TAR complexes. (**B**) CCNT1- and BRD4-associated gene expression is largely preserved in CCNT1.C261Y cells. Heat maps displaying the log_2_FC of CCNT1 (*n* = 96, left) or BRD4 (*n* = 93, right) associated genes in the C261Y clones compared to wild type. Individual genes sharing positive (red) or negative (blue) log_2_FC values were color-coded (adjusted *P*-value (P*) ≤ 0.05 (DESeq2) in both C1 and C2 cell lines). Diamond symbols denote differentially expressed genes with log_2_FC of >1 or <−1.

Analogous to HIV-1 Tat, the cellular bromodomain-containing protein 4 (BRD4) recruits p-TEFb to cellular promoters to exert control at a transcription elongation step (Fig. 7A) (67), with Tat thought to compete with BRD4 for p-TEFb binding during HIV-1 infection (67, 68). Therefore, we surveyed the published literature to compile lists of experimentally defined genes reported to be regulated by either CCNT1 (69 – 71) or BRD4 (72 – 79) and asked if either of these gene sets were more likely to be linked to the C261Y modification (Fig. 7B), using the same low stringency statistical threshold described above. Based on these criteria, only two potential CCNT1-associated genes (DTX4 and PCOLCE2) and two BRD4-associated genes (MCTP2 and IL15RA) were differentially expressed greater than twofold in both CCNT1.C261Y cell clones. Accordingly, the vast majority of genes associated with BRD4 or CCNT1 were unaffected in CCNT1.C261Y cells, further supporting the conclusion that, unlike Tat-dependent viral gene activation, cellular gene regulation in Jurkat T cells is largely unaffected by CCNT1 C261Y modification.

## DISCUSSION

In this study, we engineered human T cells using CRISPR/Cas9 genome editing to test the hypothesis that minor and “rodent-inspired” gene modifications can create a human T cell environment exhibiting intrinsic resistance to HIV-1 early and/or late-stage viral gene expression. We provide proof of concept for manifesting a block to Tat-dependent HIV-1 gene expression in human Jurkat T cells by editing a relevant species-specific locus (C261) encoded within the endogenous *CCNT1* gene. Furthermore, we validated the strength and specificity of the Tat block by isolating two HIV-1-refractory cell clones (C1 and C2) that contained prescribed, homozygous knock-in edits encoding a single nonsynonymous mutation in endogenous *CCNT1* resulting in the C261Y substitution.

Because our study relied heavily on measuring changes to HIV-1 Tat- and/or Rev-dependent gene expression in infected cell populations, we created the E-R-/2FP dual reporter assay to rapidly detect and measure defects to Tat- and Rev-dependent gene expression. In Fig. 1, we validated this assay by screening non-human mammalian cell lines predicted to pose genetic blocks to HIV-1 Tat and Rev activity (38, 80). As expected, rodent cell lines were largely refractory to HIV-1 Tat and/or Rev function. However, that Rat1 and CHO cells exhibited substantial early, Rev-independent viral gene expression was surprising considering that they encode the non-permissive CCNT1 tyrosine residue at the 261-equivalent position. The competency for early viral gene expression in these cell lines raises the possibility that there are other cell-intrinsic factors that overcome or offset the HIV-refractory effects of the Y261 allele, at least in some cell types. Cat (CRFK) cells are also of interest to us going forward because they supported Rev function despite the cat XPO1 “patch” domain differing from that of the human ortholog at two of the three known relevant species-specific residues.

Recent advances in precision CRISPR/Cas9 genome editing provided us with the means to investigate the potential to introduce species-specific Tat and Rev blocks into human T cells. Unlike HIV-refractory ∆32 *CCR5* alleles, the C261Y and P411T/M412V/F414S substitutions we engineered had not been documented in primates. Thus, at the outset, it was unclear whether human cells would tolerate these changes. Indeed, *CCNT1* and *XPO1* have only experienced modest changes throughout mammalian speciation, with the mouse and human orthologs of CCNT1 and XPO1 exhibiting 89% and 98% amino acid identity, respectively. Considering such strong negative selection, the significance of these HIV-relevant species-specific residues to basic cellular functions (if any) remains unknown. Using our CRISPR/Cas9 knock-in designs, we successfully achieved targeted editing of *CCNT1*, observing viral gene repression consistent with disruption of Tat function in viable cell populations. We also readily isolated seamlessly edited cell clones encoding exclusively CCNT1.C261Y, allowing us to confirm unequivocally that the modification is tolerated in this context. However, attempts to isolate comparably edited homozygous XPO1.P411T/M412V/F414S cells were not successful, suggesting that these modifications are deleterious.

Using the CCNT1.C261Y cell clones, we confirmed that the C261Y modification imposed a severe block specific to Tat-dependent viral gene expression rescued by exogenous delivery of wild-type CCNT1 and potently inhibited spreading HIV-1 replication (Fig. 3 and 4). Given the apparent tolerance and anti-HIV effects of the C261Y substitution in human T cells, we considered this knock-in approach in the context of a “block-and-lock” functional cure scenario (81), employing a latency reporter system (60) to demonstrate that the CCNT1.C261Y cells remain susceptible to HIV-1 infection but are markedly refractory to drug-induced viral reactivation (Fig. 5). Akin to this block, a recently described small molecule, didehydro-cortistatin A (dCA) has shown promise as a viral transcription inhibitor disrupting Tat/TAR interactions (82). Valente and colleagues recently isolated dCA-resistant HIV-1 clones with dCA resistance mapped to mutations in the viral LTR, *nef* or *vpr* genes; all of which were suggested to promote basal, Tat-independent transcription of viruses that then lost their ability to enter viral latency (83). To date, we have no indication of viral resistance to the CCNT1 C261Y modification in virus replication assays (Fig. 4). As such, we propose that CCNT1.C261Y cell lines will serve as a useful platform for longitudinal studies of latency in the context of HIV-1 LTR silencing and the net potential for LRA-driven, Tat-independent infectious virion production (84).

In Fig. 6, we showed that human T cells bearing CCNT1.C261Y limit the activity of not only HIV-1 Tat but also the Tat proteins of HIV-2 and SIV. These results emphasize how fundamental the Tat-C261 interaction must be to the replicative success of primate lentiviruses and suggest that any intervention strategy that targets the crucial CCNT1.C261 residue will be broad-spectrum, likely affecting all HIV-1 and HIV-2 strains and subtypes. Moreover, because this mechanism extends to SIV, the block may be testable in non-human primate model systems. Interestingly, a subset of non-primate lentiviruses is thought to be less dependent on CCNT1 C261 (e.g., bovine immunodeficiency virus) (85, 86) so that additional comparative structure-function studies of Tat-CCNT1 interactions seem warranted.

Aside from its crucial role in HIV-1 Tat function, little is known regarding the relevance, if any, of CCNT1 residue 261 to cellular processes; a key consideration should this protein domain be targeted in the context of antiviral strategies. CCNT1.C261Y clones exhibited no significant changes to rates of cell proliferation or basal *CCNT1* expression (Fig. 5), confirming that the C261Y modification is not profoundly detrimental, at least in cell lines. In Fig. 6, we further investigated the C261Y modification with experiments designed to interrogate endogenous p-TEFb complexes and their response to stimuli and performed a comprehensive transcriptomic analysis of CCNT1.C261Y cell lines. CDK9 co-IP experiments indicated that the steady-state levels and interactions between CCNT1 and CDK9 were unperturbed relative to the parental cells. *CCNT1* knock-out cells and mice depleted of CCNT1 have been reported with notable changes to the expression of *CDK9* and other transcription factors (87, 88). However, our glycerol gradient analysis indicated similar expression of CDK9 for both wild-type *CCNT1* and *CCNT1.C261Y* cells, as well as successful release of CDK9 from high-molecular-weight complexes in both cell backgrounds in response to DRB treatment (65, 66). Further, transcriptomic analysis using RNA-seq demonstrated that the majority of CCNT1- and BRD4-dependent genes were unperturbed in the *CCNT1.C261Y* background. Taken together, the effects of *CCNT1.C261Y* in these cell lines were largely virus-targeted, being far more severe on Tat-dependent gene expression relative to any subset of cellular genes. That said, potential functional roles for CCNT1 C261 in primary human lymphocytes and *in vivo*, if any, are pending investigation.

It remains highly interesting to us and unexplained why rodents code for variation in these otherwise conserved *CCNT1* and *XPO1* genes, with these variations prohibiting HIV-1 gene expression in a synergistic fashion. The functional consequences of this constellation of polymorphisms on modern lentiviruses including not only HIV-1 but also HIV-2 and SIV_agm_ (Fig. 4) are remarkable. We propose that harnessing these species-specific polymorphisms to engineer cell-intrinsic resistance barriers to Tat (and potentially Rev, if not both in combination) in human T cells will provide targeted means to study these selective pressures and should also be considered in the context of functional-cure-based antiviral strategies.

## MATERIALS AND METHODS

### Cell culture

Human T lymphocyte (Jurkat E6-1), human embryonic kidney (293T), human cervical carcinoma (HeLa), mouse fibroblast (3T3), and CHO cell lines were obtained from the American Type Culture Collection (ATCC). African green monkey osteosarcoma (Cos7), chicken fibroblast (DF-1), cat kidney (CRFK), mouse fibroblast (L cells, and M. dunni), and mouse myoblast (C2C12) cell lines were a kind gift of Dr. Michael Malim (King’s College London). The Rat fibroblast (Rat1) cell line was a kind gift of Dr. Robert Kalejta (University of Wisconsin-Madison). The chimpanzee skin fibroblast (WES) cell line was a kind gift of Dr. Beatrice Hahn (University of Pennsylvania). The TZM-bl reporter cell line was a kind gift of Dr. John Kappes and Dr. Xiaoyun Wu and obtained from the HIV Reagent Program (NIH). CHO, Jurkat, and Jurkat-derived cell lines were cultured in Roswell Park Memorial Institute (RPMI) 1640 supplemented to 10% fetal bovine serum (FBS), 1% penicillin-streptomycin, and 1% l-glutamine (Sigma). All other established cell lines were cultured in Dulbecco’s Modified Eagle’s Medium supplemented to 10% FBS, 1% L-glutamine, and 1% penicillin-streptomycin. Cell cultures were maintained at 37°C and 5% CO_2_ in a humidified incubator.

### Plasmids

The E-R-/2FP dual reporter virus construct is derived from the E-R-/mCherry construct described elsewhere (89). Using overlapping PCR and standard molecular cloning procedures, cDNA sequences encoding the mVenus fluorescent protein with flanking glycine-rich linker segment at the 5′-(GGSGGTR) and 3′-(TRGGSGG) ends were fused in frame with the HIV-1 *gag* sequence between matrix and capsid coding regions (90). E-R-Tat-/2FP was generated by overlapping PCR using the primers 5′-CCT AGA CTA GAG CCC TGG AAG CAT CCA TGA AGT TAA CCT AAA ACT G-3′ and 5′-GGC TCT AGT CTA GGA TCT ACT GGC TCC GTT TCT TGC TC-3′. E-R-Rev-/2FP was generated by subcloning the modified Rev coding region from E-R-Rev-/YFP described elsewhere (91). The lentiviral packaging plasmid psPax2 was a kind gift from Dr. Didier Trono (plasmid #12260, Addgene). The pVSV-G expression construct is described elsewhere (92). Retroviral vector plasmids encoding *CCNT1* variants were generated by subcloning modified *CCNT1* cDNA sequences fused to cDNA encoding the FLAG-epitope tag (DYKDDDDK) into the pELE88 vector (80) using BglII and NotI restriction enzyme sites. Retroviral vector plasmids encoding GFP or CCR5 were generated by subcloning cDNAs into MIGR1-derived vectors (93) using vector-encoded BglII and XhoI restriction enzyme sites. The retroviral packaging plasmid pMD.GagPol was a kind gift from Dr. Richard Mulligan. For the Tat/LTR reporter assay, the construction of the *gaussia* luciferase (gLuc) HIV-1 LTR reporter construct is described elsewhere (55). The pEV280 Tat-FLAG expression construct was a kind gift of Dr. Melanie Ott. Additional plasmids used for experimental controls include pmCherry-C1 (Clontech Laboratories Inc., Mountain View, CA, USA), cpTK-Cluc (New England Biolabs, Ipswich, MA, USA), pBluescript (Stratagene, La Jolla, CA, USA), and pSEAP (Clontech Laboratories Inc.). Plasmids encoding the infectious molecular clones of HIV-1, pCH106.c/2633, pRHPA.c/2635, and pSUMA.c/2821 were kind gifts of Dr. John Kappes and Dr. Christina Ochsenbauer and obtained from the HIV Reagent Program (NIH). The R7GEmC reporter virus (60) was a kind gift of Dr. Vincenzo Calvanese and Dr. Eric Verdin and was obtained from the HIV Reagent Program (NIH). The GFP-encoding and *env*-deficient HIV-1.NL4-3, HIV-2.ROD, and SIV_agm_.Tan-1 reporter virus clones are described elsewhere (58, 59) and were a kind gift of Dr. Paul Bieniasz.

### Virus preparation

To prepare virus stocks, 1 × 10^6^ 293T producer cells were plated in 10 cm tissue culture treated dishes. Twenty-four hours post plating, cells were transfected using polyethylenimine (catalog no. 23966. Polysciences, Inc., Warrington, PA, USA) followed by culture medium exchange 4 h post transfection. Forty-eight hours post transfection, cell culture supernatants were 0.45 μm filtered, aliquoted, and stored at −80°C. For E-R-/2FP reporter virus stocks, plasmid mixes contained 4 µg E-R-/2FP variant, 4 µg psPax2, and 1 µg pVSV-G. For R7GeMC reporter virus stocks, plasmid mixes contained 9 µg R7GeMC and 1 µg pVSV-G. For GFP-encoding primate lentivirus reporter stocks, plasmid mixes contained 9 µg each E- GFP vector (HIV-1.NL4-3, HIV-2.ROD, or SIV_agm_.Tan) and 1 µg pVSV-G. For MLV-based retroviral vector stocks encoding GFP, CCNT1, or CCNT1.C261Y, plasmid mixes contained 4 µg each retroviral vector plasmid, 4 µg pMD.GagPol, and 1 µg pVSV-G. For replication-competent HIV-1 virus stocks, 10 µg plasmid encoding each infectious HIV-1 molecular clone was used. When necessary, reporter virus stocks were titrated on Jurkat E6-1 cells to establish normalized infectious doses. Replication-competent HIV-1 stocks were normalized by p24^Gag^ levels determined by ELISA (PerkinElmer Life Sciences, Inc., Boston, MA, USA).

### E-R-/2FP infection assays

For infections with suspension cell lines, 1 × 10^6^ cells were plated in each well of a 6-well dish and infected immediately with equivalent doses of E-R-/2FP virus stock. For infections with adherent cell lines, the indicated cell line was plated to 30%–50% confluency in each well of a 6-well dish and infected 24 h post plating as above. Nevirapine stock was obtained from the HIV Reagent Program (NIH) and added immediately prior to infection to a final concentration of 1 µM where indicated. Forty-eight hours post infection, cells were harvested, washed in PBS, fixed with 4% reconstituted paraformaldehyde, and analyzed by flow cytometry. Flow cytometry was performed using either a LSRII (BD Biosciences, Mississauga, ON, Canada) or an Attune NxT (Thermo Fisher Scientific, Waltham, MA, USA). Flow cytometry data analysis was performed using FlowJo software (FlowJo, LLC, Ashland, OR, USA). For CCNT1 rescue experiments, 5 × 10^5^ cells were transduced by spinoculation (94) with retroviral vectors encoding CCNT1, CCNT1.C261Y, or vector only control in the presence of 5 µg/mL polybrene (Sigma) prior to plating. Following transduction, cell cultures were diluted 1:1 with fresh culture medium volume, plated in 6-well tissue culture-treated dishes, and cultured for 3 days. Cells were then infected, harvested, and analyzed as described above.

### Genome engineering of Jurkat E6-1 cells

Alt-R recombinant S.p. Cas9 nuclease-3NLS (IDT, Integrated DNA Technologies, Coralville, IA, #1074181), custom Alt-R CRISPR-Cas9 crRNAs (IDT), ATTO-550 labeled Alt-R tracrRNA (IDT, #1075928), and Alt-R Cas9 electroporation enhancer reagents were prepared, and ribonucleoprotein (RNP) complex assembly was performed according to the manufacturer’s instructions. *CCNT1* crRNA : 5′-TGC TTC ATT GCA GGC ATG CG-3′. *XPO1* crRNA : 5′-TCT GTT ACC TTG AAT AAC AT-3′. The indicated 119-nt single-stranded oligodeoxynucleotide (ssODN donor template for homology-directed repair was custom prepared (Sigma) and resuspended in TE buffer. *CCNT1* ssODN donor template: 5′-GTG TTT TTT TAT TTA GTA AAT TAC CTA AGT AAA GAG ATG CTA TTT GCT TCA TTG CAG GCG TAC GAA GCT GCC AAG AAA ACA AAA GCA GAT GAC CGA GGA ACA GAT GAA AAG ACT TCA GA-3′. *XPO1* ssODN donor template: 5′-TGC TTT CTG GAA GTC AAC ATT TTG ATG TTC CTC CCA GGA GAC AGC TGT ATT TGA CTG TGT TAT CAA AGG TAA CAG AGC GGT TGG TTG AGT GTT CTT CCT GTT GCA TAC TGT GGT TTT GA-3′. RNP complexes and ssODNs were delivered to Jurkat E6-1 cells using the Neon Transfection System and Neon Transfection 10 μL Kit (Thermo Fisher Scientific) according to manufacturer’s instructions. Electroporation parameters were 1,600 V, 10 ms pulse width, 3 pulses, and cells were cultured post-electroporation in antibiotic-free media (RPMI 1640 supplemented with 10% FBS). Twenty-four hours post electroporation, cells were bulk-sorted by FACS to isolate ATTO-550 positive cells in antibiotic-replete media (RPMI 1640 supplemented with 10% FBS and 1% penicillin-streptomycin-l-glutamine). The Jurkat E6-1 cell line was selected for gene knock-in editing because the chromosomal regions encoding *CCNT1* and *XPO1* were reportedly normal as determined by karyotype analyses (ATCC).

### CRISPR knock-in screening

Cells from each CRISPR treatment were pelleted, washed in PBS, and resuspended in 10 μL of 1× Green GoTaq Flexi Buffer (Promega, Madison, WI, USA) and stored at −80°C. Frozen cell samples were thawed and subjected to proteinase K treatment (65°C for 1 h; 95°C for 15 min; 4°C hold) to yield genomic DNA template mixture. During hold step, 40 µL reagent mix containing 1× Green GoTaq Flexi Buffer, 0.5 µM each screening primer, 0.5 µM dNTP mix, 1.5 mM MgCl_2_, and 2.5 U GoTaq DNA polymerase (GoTaq Green Flexi Kit, Promega) was added to 10 µL genomic DNA template mixture yielding 50 µL total reaction volume. Target-dependent screening primers are as follows: *CCNT1* forward screening primer, 5′-TGA GAT TAG AAG TAG GCT TGA GAG G-3′; *CCNT1* reverse screening primer, 5′-GCT AAA TTC TCA CTA GTC CGA TGA C-3′; *XPO1* forward screening primer, 5′-TTC TCT CCT CTG TGA TGG TAC ATT T-3′; *XPO1* reverse screening primer, 5′-TCA AGA TTG TAG TGA GCT ATG ACC A-3′. Optimized PCR cycling conditions yielded 1 kb-sized PCR amplicons for each target. Where indicated, prepared PCR amplicons were submitted for Sanger sequencing (Functional Biosciences, Madison, WI, USA) for analysis. Sequencing chromatograms were prepared using SnapGene software (GSL Biotech, San Diego, CA, USA). Restriction enzyme digests of PCR amplicons were carried out following the manufacturer’s recommended reaction composition with BsiWI or SacI for *CCNT1* or PvuII or XbaI for *XPO1*. Predicted *CCNT1* SacI digestion products: 804 and 196 bp. Predicted *CCNT1* BsiWI digestion products: 712 and 288 bp. Predicted *XPO1* XbaI digestion products: 679 and 298 bp. Predicted *XPO1* PvuII digestion products: 497 and 480 bp.

### E-R-/2FP FACS analysis

For infections of heterogeneous populations of CRISPR-treated Jurkat cells, equivalent numbers of unfixed and infected cells from each gate (>10,000 per gate; gate scheme shown in Fig. 2C) were analyzed by flow cytometry and sorted by FACS using a FACSAria cell sorter (BD Biosciences) instrument. Bulk-sorted cells were screened for CRISPR knock-in as described above. Corresponding flow cytometry measurements were analyzed as described above.

### SDS-PAGE and immunoblotting

To measure endogenous CCNT1 protein levels, equivalent numbers of each Jurkat cell line were pelleted, washed with PBS, and lysed using radioimmunoprecipitation assay buffer (10 mM Tris-HCl [pH 7.5], 150 mM NaCl, 1 mM EDTA, 0.1% sodium dodecyl sulfate [SDS], 1% Triton X-100, 1% sodium deoxycholate) containing complete protease inhibitor cocktail (Roche). Cell lysates were prepared for immunoblot by sonication and clarified supernatants were boiled with 2× dissociation buffer (62.5 mM Tris-HCl [pH 6.8], 10% glycerol, 2% SDS, 10% β-mercaptoethanol) at a 1:1 ratio prior to polyacrylamide gel electrophoresis (PAGE) and immunoblot. Prepared cell lysates were subjected to SDS-PAGE using 10% polyacrylamide gels and a conventional Tris-glycine buffering system. Following electrophoresis, resolved lysates were transferred to 0.2 µm pore size nitrocellulose membranes (GE Healthcare) for immunoblot. Nitrocellulose membranes were blocked in blocking solution (PBS containing 0.1% Tween-20 (PBS-T) and reconstituted non-fat dry milk at a 2% final concentration). Following incubation in the blocking solution, membranes were incubated in primary antibody solutions consisting of fresh blocking solution containing CCNT1 antisera (Santa Cruz Biosciences, Dallas, TX, USA, sc-398695; Cell Signaling Technologies, Danvers, MA, USA D1B6G), CDK9 antisera (Bethyl Laboratories, Montgomery, TX, USA, A303-493A), or HSP90 antisera (Santa Cruz Biosciences, sc-7947; sc-13119). Following incubation in primary antibody solution, membranes were washed three times with PBS-T and incubated in secondary antibody solutions consisting of fresh blocking solution containing either anti-mouse or anti-rabbit secondary antisera conjugated to either IRDye680 or IRDye800 (LI-COR Biosciences, Lincoln, NE, USA) infrared fluorophores. Following incubation in secondary antibody solution, membranes were washed three times in PBS-T and analyzed by quantitative immunoblot using an Odyssey Fc instrument (LI-COR Biosciences).

### Cell proliferation assay

A total of 5 × 10^4^ cells from each Jurkat line was plated in each well of a 12-well tissue culture treated dish containing 1 mL of cell culture medium. At 4 and 6 days post plating, cultures for each cell line were resuspended, diluted, and mixed at 1:1 concentration with a 0.4% Trypan blue (Sigma) solution. Cells lacking Trypan blue uptake were counted manually using a hemocytometer.

### Transient HIV-1 LTR reporter assays

For each Jurkat cell line, 5 × 10^5^ cells were transfected using the Neon electroporation system using the electroporation parameters described above per the manufacturer’s instructions. Plasmid mixtures contained 75 ng of HIV-1 LTR reporter constructs *gaussia* luciferase (gLuc) and nanoluciferase (nLuc, Promega), 250 ng pmCherry-C1 plasmid, 250 ng pTK-cLuc plasmid, 1,200 ng CCNT1 or control plasmid (pBluescript or pSEAP), and 25 ng pEV280 Tat expression plasmid or control carrier DNA. Total DNA concentrations were maintained at 2,500 ng by vector control plasmids or calf thymus DNA. Twenty-four hours post transfection, 10 µL culture media was removed, diluted with 40 µL PBS, and assayed for secreted *gaussia* luciferase (gLuc) activity by injecting 30 µL coelenterazine solution (Promega) followed by 1.6 s incubation period and a luminescence integration read time of 1.0 s. Secreted *cypridina* luciferase (cLuc) activity from the internal control plasmid was similarly measured using the *cypridina* luciferase kit (New England Biolabs) according to the manufacturer’s instructions. Fold activation was calculated as the ratio of gLuc:cLuc signals in each well compared to the average of the pBluescript and pSEAP control transfections.

### HIV-1 infection assays

A total of 2.5 × 10^6^ cells from each Jurkat cell line was plated in each well of a 48-well plate and infected with HIV variants, NL4-3 or IIIB, at a multiplicity of infection of 0.5. The following day, the inoculum was removed, cells were washed twice with PBS and then replenished with fresh culture medium, and maintained at normal culture conditions. At 4 and 10 days post infection, cells and supernatant were collected for viral RNA quantification, viral outgrowth assays, and flow cytometry staining analysis.

### Viral RNA quantification

Viral RNA measurements were performed using the method described previously (95). From 100 µL of supernatant collected at each time point, viral RNA was extracted using the QIAamp viral RNA mini kit (Qiagen, Hilden, Germany) following the manufacturer’s protocol. SYBR green real-time PCR assay was carried out in a 20-µL PCR mixture volume consisting of 10 µL of 2× Quantitect SYBR green RT-PCR Master Mix (Qiagen) containing HotStarTaq DNA polymerase, 0.5 µL of 500 nM each oligonucleotide primer, 0.2 µL of 100× QuantiTect RT Mix (containing Omniscript and Sensiscript RTs), and 8 µL of RNA extracted from samples or standard HIV-1 RNA (from 5 × 10^5^ to 5 copies per 1 mL). Highly conserved sequences on the gag region of HIV-1 were chosen, and specific HIV-1 gag primers were selected. The sequences of HIV-1 gag primers are 5′-CAA TGG CAG CAA TTT CAC CA-3′ and 5′-GAA TGC CAA ATT CCT GCT TGA-3′. Amplification was done in an Applied Biosystems 7,500 real-time PCR system (Applied Biosystems, Waltham, MA, USA), and it involved activation at 45°C for 15 min and 95°C for 15 min followed by 40 amplification cycles of 95°C for 15 s, 60°C for 15 s, and 72°C for 30 s. For the detection and quantification of viral RNA, the real-time PCR of each sample was compared with threshold cycle value of a standard curve.

### Viral outgrowth assay in TZM-bl cells

Twenty-four hours prior to infection, 20,000 TZM-bl cells were plated in each well of a 48-well tissue culture-treated plate. The cells were incubated with 10 µL supernatant collected from infected Jurkat cell cultures for overnight incubation at 37°C. The following day, infected cells were washed and fresh culture media was added. Forty-eight hours post infection, luciferase activity in cell lysates was measured using a luciferase assay kit, following the manufacturer’s protocol (Promega).

### p24^Gag^ staining

HIV-infected cells for each Jurkat cell lines were permeabilized using the Cytofix/Cytoperm Fixation/Permeabilization Kit (BD Biosciences) and stained intracellularly using PE-conjugated mouse anti-p24 mAb (clone KC57; Beckman Coulter, Brea, CA, USA, 1:100 dilution). Flow cytometry measurements were performed using a FACSCalibur (BD Biosciences) and data analysis was performed as above.

### HIV-1 replication curves

A total of 5 × 10^6^ cells from each Jurkat cell line was infected with 10 ng p24^Gag^-equivalent volumes for the indicated HIV-1 variants in 6 mL antibiotic-replete RPMI. Twenty-four hours post infection, cultures were pelleted, washed three times with 1× PBS, and resuspended in fresh culture medium. Each 6 mL culture was maintained under normal culture conditions and split by removal of 4 mL culture (media containing cells) and refreshed with an equal volume of fresh media every 3 days. Where indicated and prior to infection, cells were transduced with a *CCR5*-encoding retroviral vector to confer susceptibility R5-tropic HIV-1 variants. Harvested cultures were processed by 0.45 μm filtration to remove cells and debris and aliquots were stored at −80°C. As previously described, p24^Gag^ levels were quantified by ELISA periodically (every 1–2 weeks) to monitor supernatant virus particle levels over time.

### R7GeMC infection assays

A total of 1 × 10^6^ cells was plated in each well of a 6-well dish and infected immediately with equivalent doses of R7GeMC virus stock. Twenty-four hours post infection, cells were pelleted and washed thrice in PBS and then resuspended in culture medium containing phorbol 12-myristate 13-acetate (PMA, Sigma) or tumor necrosis factor alpha (EMD Millipore, 80054-834) at 10 ng mL^−1^ or vehicle control (DMSO) where indicated. Two days post treatment, cells were harvested, processed, and analyzed by flow cytometry as described above.

### Retroviral GFP reporter assays

A total of 1 × 10^6^ cells was plated in each well of a 6-well dish and infected immediately with equivalent doses of the indicated GFP-encoding reporter virus stock. Twenty-four hours post infection, cells were harvested, processed, and analyzed by flow cytometry as described above.

### Glycerol gradients

A total of 5 × 10^7^ cells for each Jurkat cell line was incubated in the presence of 10 µM 5,6-dichlorobenzimidazole 1-β-D-ribofuranoside (DRB, Sigma) or DMSO vehicle control for 2 h under normal culture conditions. Cells were then pelleted, washed with chilled PBS, and lysed in 0.5 mL glycerol gradient lysis buffer (GGLB; 150 mM NaCl, 10 mM KCl, 10 mM MgCl_2_, 10 mM HEPES [pH 7.5], 2 mM β-mercaptoethanol, 0.5% NP-40, 1× ProteoBlock protease inhibitor cocktail [Fermentas, Hanover, MD]) for 10 min on ice. Cell lysates were centrifuged to pellet nuclei (20,000 × *g* for 5 min at 4°C) and supernatants were layered atop a 5%–45% glycerol gradient prepared with GGLB. Samples were centrifuged (190,000 × I for 16 h at 4°C) using a TLS-55 rotor. Following centrifugation, fractions were pipetted into fresh tubes, prepared for SDS-PAGE and immunoblotting, and analyzed as described above. Fractions 6–8 (active) and 11–12 (inactive) were used to determine DRB responses by calculating fold-induction over untreated controls.

### Co-immunoprecipitation

An amount of 5 × 10^6^ Tat-expressing cells for each Jurkat cell line was lysed in 500 µL ice cold Tris buffered saline NP-40 (TBSN) (150 mM NaCl, 50 mM TRIS [pH 7.5], 1 mM EDTA, 2 mM β-mercaptoethanol, 0.5% NP40, 1× HALT protease inhibitor cocktail [Invitrogen, Carlsbad, CA]) buffer. Cells were lysed on ice for 10 min, nuclei were pelleted (20,000 × I for 10 min at 4°C), and supernatant was transferred to a new tube. Fifty microliters (10%) of the input material was removed for SDS-PAGE. The remaining sample was immunoprecipitated with anti-CCNT1 antibody (Bethyl Laboratories, A303-499A) or non-specific normal rabbit IgG (Cell Signaling Technologies, 2,729S) in the presence of 2,000 gel units micrococcal nuclease (New England Biolabs) for 1 h. Fifty microliters of magnetic protein A/G beads (Pierce, 88803) was added, and the samples were incubated for an additional hour. The samples were washed thrice in TBSN, and the beads were resuspended in 50 µL SDS-PAGE loading buffer. The samples were boiled, and equivalent volumes of input and IP elution were separated by SDS-PAGE and immunoblotted with mouse anti-CDK9 (Santa Cruz Biosciences, sc-13130) and mouse anti-CCNT1 (Santa Cruz Biosciences, sc-271348) as described above.

### Transcriptome analysis

For each Jurkat cell line, 1.5 × 10^6^ cells in 10 mL fresh culture medium were plated in T75 tissue culture-treated flasks and maintained under normal culture conditions. Forty-eight hours post plating, cells were pelleted from suspension (400 × *g* for 5 min at 4°C) and delicately resuspended in 1 mL chilled PBS and transferred to 1.5 mL microcentrifuge tube. Cell suspensions were pelleted (400 × *g* for 5 min at 4°C), and clarified PBS was aspirated. Cell pellets were stored at −80°C. Total RNA was extracted from cell pellets of three biological replicates (i.e., time-separated cultures) using RNeasy Plus Mini Kit (Qiagen) with genomic DNA eliminator columns and resuspended in nuclease-free water according to the manufacturer’s instructions. RNA extracts were stored at −80°C prior to submission to the University of Wisconsin-Madison Biotechnology Center for quality control assessment, library preparation, and sequencing. RNA sample concentrations and quality assessments were measured using a NanoDrop One spectrophotometer (software version 1.4.0) and an Agilent 2100 Bioanalyzer (G2939A; firmware: C.01.069; Assay: Eukaryote Total RNA Nano). Poly(A)-enriched mRNA-sequencing libraries were prepared using an Illumina TruSeq stranded mRNA Library Prep Kit (Illumina, San Diego, CA, USA) according to the manufacturer’s instructions. Samples were loaded onto a MiSeq NanoCell (Illumina), and paired-end RNA sequencing was performed on a NovaSeq 6,000 sequencing platform (Illumina). Sequencing was carried out with a read depth target of approximately 30 million reads per sample. Sequencing reads were mapped to human genome assembly hg38 using HISAT2 (96) (version 2.0.5). Annotated feature count tables were prepared using the htseq-count tool within HTSeq (97) (version 0.6.0). DGE analyses were performed using DESeq2 (version 1.26.0) (98). Computational resources for RNA sequencing analysis were kindly provided by the Bioinformatics Resource Center at the University of Wisconsin—Madison.

## Data Availability

RNA-seq data have been deposited to the Gene Expression Omnibus (https://www.ncbi.nlm.nih.gov/geo) and can be accessed via GSE209745.

## References

[1] Liu R , Paxton WA , Choe S , Ceradini D , Martin SR , Horuk R , MacDonald ME , Stuhlmann H , Koup RA , Landau NR . 1996. Homozygous defect in HIV-1 coreceptor accounts for resistance of some multiply-exposed individuals to HIV-1 infection. Cell 86:367–377. doi:10.1016/s0092-8674(00)80110-5 8756719

[2] Samson M , Libert F , Doranz BJ , Rucker J , Liesnard C , Farber CM , Saragosti S , Lapoumeroulie C , Cognaux J , Forceille C , Muyldermans G , Verhofstede C , Burtonboy G , Georges M , Imai T , Rana S , Yi Y , Smyth RJ , Collman RG , Doms RW , Vassart G , Parmentier M . 1996. Resistance to HIV-1 infection in caucasian individuals bearing mutant alleles of the CCR-5 chemokine receptor gene. Nature 382:722–725. doi:10.1038/382722a0 8751444

[3] Huang Y , Paxton WA , Wolinsky SM , Neumann AU , Zhang L , He T , Kang S , Ceradini D , Jin Z , Yazdanbakhsh K , Kunstman K , Erickson D , Dragon E , Landau NR , Phair J , Ho DD , Koup RA . 1996. The role of a mutant CCR5 allele in HIV–1 transmission and disease progression. Nat Med 2:1240–1243. doi:10.1038/nm1196-1240 8898752

[4] Hütter G , Nowak D , Mossner M , Ganepola S , Müssig A , Allers K , Schneider T , Hofmann J , Kücherer C , Blau O , Blau IW , Hofmann WK , Thiel E . 2009. Long-term control of HIV by CCR5 delta32/delta32 stem-cell transplantation. N Engl J Med 360:692–698. doi:10.1056/NEJMoa0802905 19213682

[5] Allers K , Hütter G , Hofmann J , Loddenkemper C , Rieger K , Thiel E , Schneider T . 2011. Evidence for the cure of HIV infection by CCR5Δ32/Δ32 stem cell transplantation. Blood 117:2791–2799. doi:10.1182/blood-2010-09-309591 21148083

[6] Gupta RK , Abdul-Jawad S , McCoy LE , Mok HP , Peppa D , Salgado M , Martinez-Picado J , Nijhuis M , Wensing AMJ , Lee H , Grant P , Nastouli E , Lambert J , Pace M , Salasc F , Monit C , Innes AJ , Muir L , Waters L , Frater J , Lever AML , Edwards SG , Gabriel IH , Olavarria E . 2019. HIV-1 remission following CCR5Δ32/Δ32 haematopoietic stem-cell transplantation. Nature 568:244–248. doi:10.1038/s41586-019-1027-4 30836379 PMC7275870

[7] Gupta RK , Peppa D , Hill AL , Gálvez C , Salgado M , Pace M , McCoy LE , Griffith SA , Thornhill J , Alrubayyi A , Huyveneers LEP , Nastouli E , Grant P , Edwards SG , Innes AJ , Frater J , Nijhuis M , Wensing AMJ , Martinez-Picado J , Olavarria E . 2020. Evidence for HIV-1 cure after CCR5Δ32/Δ32 allogeneic haemopoietic stem-cell transplantation 30 months post analytical treatment interruption: a case report. Lancet HIV 7:e340–e347. doi:10.1016/S2352-3018(20)30069-2 32169158 PMC7606918

[8] Kordelas L , Verheyen J , Beelen DW , Horn PA , Heinold A , Kaiser R . 2014. Shift of HIV tropism in stem-cell transplantation with CCR5 delta32 mutation. N Engl J Med 371:880–882. doi:10.1056/NEJMc1412279 25162903

[9] Glass WG , McDermott DH , Lim JK , Lekhong S , Yu SF , Frank WA , Pape J , Cheshier RC , Murphy PM . 2006. CCR5 deficiency increases risk of symptomatic west Nile virus infection. J Exp Med 203:35–40. doi:10.1084/jem.20051970 16418398 PMC2118086

[10] Kindberg E , Mickiene A , Ax C , Akerlind B , Vene S , Lindquist L , Lundkvist A , Svensson L . 2008. A deletion in the chemokine receptor 5 (CCR5) gene is associated with tickborne encephalitis. J Infect Dis 197:266–269. doi:10.1086/524709 18179389

[11] Lim JK , Louie CY , Glaser C , Jean C , Johnson B , Johnson H , McDermott DH , Murphy PM . 2008. Genetic deficiency of chemokine receptor CCR5 is a strong risk factor for symptomatic west Nile virus infection: a meta-analysis of 4 cohorts in the US epidemic. J Infect Dis 197:262–265. doi:10.1086/524691 18179388

[12] Malim MH , Bieniasz PD . 2012. HIV restriction factors and mechanisms of evasion. Cold Spring Harb Perspect Med 2:a006940. doi:10.1101/cshperspect.a006940 22553496 PMC3331687

[13] Hotter D , Kirchhoff F . 2018. Interferons and beyond: induction of antiretroviral restriction factors. J Leukoc Biol 103:465–477. doi:10.1002/JLB.3MR0717-307R 29345347

[14] Mariani R , Chen D , Schröfelbauer B , Navarro F , König R , Bollman B , Münk C , Nymark-McMahon H , Landau NR . 2003. Species-specific exclusion of APOBEC3G from HIV-1 virions by Vif. Cell 114:21–31. doi:10.1016/s0092-8674(03)00515-4 12859895

[15] Stremlau M , Owens CM , Perron MJ , Kiessling M , Autissier P , Sodroski J . 2004. The cytoplasmic body component TRIM5α restricts HIV-1 infection in old world monkeys. Nature 427:848–853. doi:10.1038/nature02343 14985764

[16] Morrow WJW , Wharton M , Lau D , Levy JA . 1987. Small animals are not susceptible to human immunodeficiency virus infection. J Gen Virol 68 (Pt 8):2253–2257. doi:10.1099/0022-1317-68-8-2253 2886549

[17] Maddon PJ , Dalgleish AG , McDougal JS , Clapham PR , Weiss RA , Axel R . 1986. The T4 gene encodes the AIDS virus receptor and is expressed in the immune system and the brain. Cell 47:333–348. doi:10.1016/0092-8674(86)90590-8 3094962

[18] Landau NR , Warton M , Littman DR . 1988. The envelope glycoprotein of the human immunodeficiency virus binds to the immunoglobulin-like domain of CD4. Nature 334:159–162. doi:10.1038/334159a0 3260352

[19] Clayton LK , Hussey RE , Steinbrich R , Ramachandran H , Husain Y , Reinherz EL . 1988. Substitution of murine for human CD4 residues identifies amino acids critical for HIV-gp120 binding. Nature 335:363–366. doi:10.1038/335363a0 2843773

[20] Dragic T , Charneau P , Clavel F , Alizon M . 1992. Complementation of murine cells for human immunodeficiency virus envelope/CD4-mediated fusion in human/murine Heterokaryons. J Virol 66:4794–4802. doi:10.1128/JVI.66.8.4794-4802.1992 1629956 PMC241307

[21] Dragic T , Litwin V , Allaway GP , Martin SR , Huang Y , Nagashima KA , Cayanan C , Maddon PJ , Koup RA , Moore JP , Paxton WA . 1996. HIV-1 entry into CD4^+^ cells is mediated by the chemokine receptor CC-CKR-5. Nature 381:667–673. doi:10.1038/381667a0 8649512

[22] Deng H , Liu R , Ellmeier W , Choe S , Unutmaz D , Burkhart M , Di Marzio P , Marmon S , Sutton RE , Hill CM , Davis CB , Peiper SC , Schall TJ , Littman DR , Landau NR . 1996. Identification of a major co-receptor for primary isolates of HIV-1. Nature 381:661–666. doi:10.1038/381661a0 8649511

[23] Atchison RE , Gosling J , Monteclaro FS , Franci C , Digilio L , Charo IF , Goldsmith MA . 1996. Multiple extracellular elements of CCR5 and HIV-1 entry: dissociation from response to chemokines. Science 274:1924–1926. doi:10.1126/science.274.5294.1924 8943208

[24] Bieniasz PD , Fridell RA , Aramori I , Ferguson SSG , Caron MG , Cullen BR . 1997. HIV‐1‐induced cell fusion is mediated by multiple regions within both the viral envelope and the CCR‐5 co‐receptor. EMBO J 16:2599–2609. doi:10.1093/emboj/16.10.2599 9184207 PMC1169871

[25] Picard L , Simmons G , Power CA , Meyer A , Weiss RA , Clapham PR . 1997. Multiple extracellular domains of CCR-5 contribute to human immunodeficiency virus type 1 entry and fusion. J Virol 71:5003–5011. doi:10.1128/JVI.71.7.5003-5011.1997 9188565 PMC191733

[26] Kuhmann SE , Platt EJ , Kozak SL , Kabat D . 1997. Polymorphisms in the CCR5 genes of African green monkeys and mice implicate specific amino acids in infections by simian and human immunodeficiency viruses. J Virol 71:8642–8656. doi:10.1128/JVI.71.11.8642-8656.1997 9343222 PMC192328

[27] Ross TM , Bieniasz PD , Cullen BR . 1998. Multiple residues contribute to the inability of murine CCR-5 to function as a coreceptor for macrophage-tropic human immunodeficiency virus type 1 isolates. J Virol 72:1918–1924. doi:10.1128/JVI.72.3.1918-1924.1998 9499044 PMC109483

[28] Browning J , Horner JW , Pettoello-Mantovani M , Raker C , Yurasov S , DePinho RA , Goldstein H . 1997. Mice transgenic for human CD4 and CCR5 are susceptible to HIV infection. Proc Natl Acad Sci U S A 94:14637–14641. doi:10.1073/pnas.94.26.14637 9405665 PMC25078

[29] Hart CE , Ou C-Y , Galphin JC , Moore J , Bacheler LT , Wasmuth JJ , Petteway SR , Schochetman G . 1989. Human chromosome 12 is required for elevated HIV-1 expression in human-hamster hybrid cells. Sci Wash 246:488–491. doi:10.1126/science.2683071 2683071

[30] Newstein M , Stanbridge EJ , Casey G , Shank PR . 1990. Human chromosome 12 encodes a species-specific factor which increases human immunodeficiency virus type 1 tat-mediated trans activation in rodent cells. J Virol 64:4565–4567. doi:10.1128/JVI.64.9.4565-4567.1990 2200890 PMC247929

[31] Alonso A , Derse D , Peterlin BM . 1992. Human chromosome 12 is required for optimal interactions between tat and TAR of human immunodeficiency virus type 1 in rodent cells. J Virol 66:4617–4621. doi:10.1128/JVI.66.7.4617-4621.1992 1602563 PMC241279

[32] Winslow BJ , Trono D . 1993. The blocks to human immunodeficiency virus type 1 tat and rev functions in mouse cell lines are independent. J Virol 67:2349–2354. doi:10.1128/JVI.67.4.2349-2354.1993 8445733 PMC240394

[33] Wei P , Garber ME , Fang SM , Fischer WH , Jones KA . 1998. A novel CDK9-associated C-type cyclin interacts directly with HIV-1 tat and mediates its high-affinity, loop-specific binding to TAR RNA. Cell 92:451–462. doi:10.1016/s0092-8674(00)80939-3 9491887

[34] Garber ME , Wei P , KewalRamani VN , Mayall TP , Herrmann CH , Rice AP , Littman DR , Jones KA . 1998. The interaction between HIV-1 tat and human cyclin T1 requires zinc and a critical cysteine residue that is not conserved in the murine Cyct1 protein. Genes Dev 12:3512–3527. doi:10.1101/gad.12.22.3512 9832504 PMC317238

[35] Bieniasz PD , Grdina TA , Bogerd HP , Cullen BR . 1998. Recruitment of a protein complex containing tat and cyclin T1 to TAR governs the species specificity of HIV-1 tat. EMBO J 17:7056–7065. doi:10.1093/emboj/17.23.7056 9843510 PMC1171053

[36] Fujinaga K , Taube R , Wimmer J , Cujec TP , Peterlin BM . 1999. Interactions between human cyclin t, tat, and the transactivation response element (TAR) are disrupted by a cysteine to tyrosine substitution found in mouse cyclin T. Proc Natl Acad Sci U S A 96:1285–1290. doi:10.1073/pnas.96.4.1285 9990016 PMC15455

[37] Trono D , Baltimore D . 1990. A human cell factor is essential for HIV-1 rev action. EMBO J 9:4155–4160. doi:10.1002/j.1460-2075.1990.tb07638.x 2249669 PMC552190

[38] Bieniasz PD , Cullen BR . 2000. Multiple blocks to human immunodeficiency virus type 1 replication in rodent cells. J Virol 74:9868–9877. doi:10.1128/JVI.74.21.9868-9877.2000 11024113 PMC102023

[39] Koito A , Shigekane H , Matsushita S . 2003. Ability of small animal cells to support the postintegration phase of human immunodeficiency virus type-1 replication. Virology 305:181–191. doi:10.1006/viro.2002.1755 12504551

[40] Zheng YH , Yu HF , Peterlin BM . 2003. Human p32 protein relieves a post-transcriptional block to HIV replication in murine cells. Nat Cell Biol 5:611–618. doi:10.1038/ncb1000 12833064

[41] Shukla RR , Marques SM , Kimmel PL , Kumar A . 1996. Human chromosome 6- and 11-encoded factors support human immunodeficiency virus type 1 rev function in A9 cells. J Virol 70:9064–9068. doi:10.1128/JVI.70.12.9064-9068.1996 8971045 PMC191013

[42] Mariani R , Rutter G , Harris ME , Hope TJ , Kräusslich HG , Landau NR . 2000. A block to human immunodeficiency virus type 1 assembly in murine cells. J Virol 74:3859–3870. doi:10.1128/jvi.74.8.3859-3870.2000 10729160 PMC111894

[43] Chen BK , Rousso I , Shim S , Kim PS . 2001. Efficient assembly of an HIV-1/MLV gag-chimeric virus in murine cells. Proc Natl Acad Sci U S A 98:15239–15244. doi:10.1073/pnas.261563198 11742097 PMC65013

[44] Coskun AK , van Maanen M , Nguyen V , Sutton RE . 2006. Human chromosome 2 carries a gene required for production of infectious human immunodeficiency virus type 1. J Virol 80:3406–3415. doi:10.1128/JVI.80.7.3406-3415.2006 16537608 PMC1440379

[45] Hübner W , Chen BK . 2006. Inhibition of viral assembly in murine cells by HIV-1 matrix. Virology 352:27–38. doi:10.1016/j.virol.2006.04.024 16750235

[46] Sherer NM , Swanson CM , Papaioannou S , Malim MH . 2009. Matrix mediates the functional link between human immunodeficiency virus type 1 RNA nuclear export elements and the assembly competency of gag in murine cells. J Virol 83:8525–8535. doi:10.1128/JVI.00699-09 19535446 PMC2738188

[47] Okada H , Zhang X , Ben Fofana I , Nagai M , Suzuki H , Ohashi T , Shida H . 2009. Synergistic effect of human Cyct1 and CRM1 on HIV-1 propagation in rat T cells and macrophages. Retrovirology 6:43. doi:10.1186/1742-4690-6-43 19435492 PMC2693497

[48] Nagai-Fukataki M , Ohashi T , Hashimoto I , Kimura T , Hakata Y , Shida H . 2011. Nuclear and cytoplasmic effects of human CRM1 on HIV-1 production in rat cells. Genes Cells 16:203–216. doi:10.1111/j.1365-2443.2010.01476.x 21251165

[49] Sherer NM , Swanson CM , Hué S , Roberts RG , Bergeron JRC , Malim MH . 2011. Evolution of a species-specific determinant within human CRM1 that regulates the post-transcriptional phases of HIV-1 replication. PLOS Pathog 7:e1002395. doi:10.1371/journal.ppat.1002395 22114565 PMC3219727

[50] Elinav H , Wu Y , Coskun A , Hryckiewicz K , Kemler I , Hu Y , Rogers H , Hao B , Ben Mamoun C , Poeschla E , Sutton R . 2012. Human CRM1 augments production of infectious human and feline immunodeficiency viruses from murine cells. J Virol 86:12053–12068. doi:10.1128/JVI.01970-12 22933280 PMC3486471

[51] Karn J , Stoltzfus CM . 2012. Transcriptional and posttranscriptional regulation of HIV-1 gene expression. Cold Spring Harb Perspect Med 2:a006916. doi:10.1101/cshperspect.a006916 22355797 PMC3281586

[52] Aligeti M , Behrens RT , Pocock GM , Schindelin J , Dietz C , Eliceiri KW , Swanson CM , Malim MH , Ahlquist P , Sherer NM . 2014. Cooperativity among rev-associated nuclear export signals regulates HIV-1 gene expression and is a determinant of virus species tropism. J Virol 88:14207–14221. doi:10.1128/JVI.01897-14 25275125 PMC4249125

[53] Booth DS , Cheng Y , Frankel AD . 2014. The export receptor Crm1 forms a dimer to promote nuclear export of HIV RNA. Elife 3:e04121. doi:10.7554/eLife.04121 25486595 PMC4360530

[54] Yue Y , Coskun AK , Jawanda N , Auer J , Sutton RE . 2018. Differential interaction between human and murine Crm1 and lentiviral rev proteins. Virology 513:1–10. doi:10.1016/j.virol.2017.09.027 29028476 PMC5914484

[55] Bruce JW , Reddington R , Mathieu E , Bracken M , Young JAT , Ahlquist P . 2013. ZASC1 stimulates HIV-1 transcription elongation by recruiting P-TEFb and TAT to the LTR promoter. PLOS Pathog 9:e1003712. doi:10.1371/journal.ppat.1003712 24204263 PMC3812036

[56] Ochsenbauer C , Edmonds TG , Ding H , Keele BF , Decker J , Salazar MG , Salazar-Gonzalez JF , Shattock R , Haynes BF , Shaw GM , Hahn BH , Kappes JC . 2012. Generation of transmitted/founder HIV-1 infectious molecular clones and characterization of their replication capacity in CD4 T lymphocytes and monocyte-derived macrophages. J Virol 86:2715–2728. doi:10.1128/JVI.06157-11 22190722 PMC3302286

[57] Bieniasz PD , Grdina TA , Bogerd HP , Cullen BR . 1999. Analysis of the effect of natural sequence variation in tat and in cyclin T on the formation and RNA binding properties of tat-cyclin T complexes. J Virol 73:5777–5786. doi:10.1128/JVI.73.7.5777-5786.1999 10364329 PMC112638

[58] Hatziioannou T , Cowan S , Goff SP , Bieniasz PD , Towers GJ . 2003. Restriction of multiple divergent retroviruses by Lv1 and Ref1. EMBO J 22:385–394. doi:10.1093/emboj/cdg042 12554640 PMC140727

[59] Hatziioannou T , Cowan S , Bieniasz PD . 2004. Capsid-dependent and -independent postentry restriction of primate lentivirus tropism in rodent cells. J Virol 78:1006–1011. doi:10.1128/jvi.78.2.1006-1011.2004 14694132 PMC368775

[60] Calvanese V , Chavez L , Laurent T , Ding S , Verdin E . 2013. Dual-color HIV reporters trace a population of latently infected cells and enable their purification. Virology 446:283–292. doi:10.1016/j.virol.2013.07.037 24074592 PMC4019006

[61] Moranguinho I , Valente ST . 2020. Block-and-lock: new horizons for a cure for HIV-1. Viruses 12:1443. doi:10.3390/v12121443 33334019 PMC7765451

[62] Zhou Q , Yik JHN . 2006. The Yin and Yang of P-TEFb regulation: implications for human immunodeficiency virus gene expression and global control of cell growth and differentiation. Microbiol Mol Biol Rev 70:646–659. doi:10.1128/MMBR.00011-06 16959964 PMC1594588

[63] Adelman K , Lis JT . 2012. Promoter-proximal pausing of RNA polymerase II: emerging roles in metazoans. Nat Rev Genet 13:720–731. doi:10.1038/nrg3293 22986266 PMC3552498

[64] Zhou Q , Li T , Price DH . 2012. RNA polymerase II elongation control. Annu Rev Biochem 81:119–143. doi:10.1146/annurev-biochem-052610-095910 22404626 PMC4273853

[65] Biglione S , Byers SA , Price JP , Nguyen VT , Bensaude O , Price DH , Maury W . 2007. Inhibition of HIV-1 replication by P-TEFb inhibitors DRB, seliciclib and flavopiridol correlates with release of free P-TEFb from the large, inactive form of the complex. Retrovirology 4:47. doi:10.1186/1742-4690-4-47 17625008 PMC1948018

[66] Nguyen VT , Kiss T , Michels AA , Bensaude O . 2001. 7Sk small nuclear RNA binds to and inhibits the activity of CDK9/Cyclin T complexes. Nature 414:322–325. doi:10.1038/35104581 11713533

[67] Yang Z , Yik JHN , Chen R , He N , Jang MK , Ozato K , Zhou Q . 2005. Recruitment of P-TEFb for stimulation of transcriptional elongation by the bromodomain protein Brd4. Mol Cell 19:535–545. doi:10.1016/j.molcel.2005.06.029 16109377

[68] Bisgrove DA , Mahmoudi T , Henklein P , Verdin E . 2007. Conserved P-TEFb-interacting domain of BRD4 inhibits HIV transcription. Proc Natl Acad Sci U S A 104:13690–13695. doi:10.1073/pnas.0705053104 17690245 PMC1959443

[69] Yu W , Wang Y , Shaw CA , Qin X-F , Rice AP . 2006. Induction of the HIV-1 tat co-factor cyclin T1 during monocyte differentiation is required for the regulated expression of a large portion of cellular mRNAs. Retrovirology 3:32. doi:10.1186/1742-4690-3-32 16764723 PMC1557533

[70] Yu W , Ramakrishnan R , Wang Y , Chiang K , Sung TL , Rice AP . 2008. Cyclin T1-dependent genes in activated CD4^+^ T and macrophage cell lines appear enriched in HIV-1 co-factors. PLOS ONE 3:e3146. doi:10.1371/journal.pone.0003146 18773076 PMC2519787

[71] Ramakrishnan R , Yu W , Rice AP . 2011. Limited redundancy in genes regulated by cyclin T2 and cyclin T1. BMC Res Notes 4:260. doi:10.1186/1756-0500-4-260 21791050 PMC3160394

[72] Yang Z , He N , Zhou Q . 2008. Brd4 recruits P-TEFb to chromosomes at late mitosis to promote G1 gene expression and cell cycle progression. Mol Cell Biol 28:967–976. doi:10.1128/MCB.01020-07 18039861 PMC2223388

[73] Mochizuki K , Nishiyama A , Jang MK , Dey A , Ghosh A , Tamura T , Natsume H , Yao H , Ozato K . 2008. The bromodomain protein Brd4 stimulates G1 gene transcription and promotes progression to S phase. J Biol Chem 283:9040–9048. doi:10.1074/jbc.M707603200 18223296 PMC2431025

[74] Liu W , Ma Q , Wong K , Li W , Ohgi K , Zhang J , Aggarwal A , Rosenfeld MG . 2013. Brd4 and JMJD6-associated anti-pause enhancers in regulation of transcriptional pause release. Cell 155:1581–1595. doi:10.1016/j.cell.2013.10.056 24360279 PMC3886918

[75] Kanno T , Kanno Y , LeRoy G , Campos E , Sun H-W , Brooks SR , Vahedi G , Heightman TD , Garcia BA , Reinberg D , Siebenlist U , O’Shea JJ , Ozato K . 2014. BRD4 assists elongation of both coding and enhancer RNAs by interacting with acetylated histones. Nat Struct Mol Biol 21:1047–1057. doi:10.1038/nsmb.2912 25383670 PMC4720983

[76] Winter GE , Mayer A , Buckley DL , Erb MA , Roderick JE , Vittori S , Reyes JM , di Iulio J , Souza A , Ott CJ , Roberts JM , Zeid R , Scott TG , Paulk J , Lachance K , Olson CM , Dastjerdi S , Bauer S , Lin CY , Gray NS , Kelliher MA , Churchman LS , Bradner JE . 2017. BET bromodomain proteins function as master transcription elongation factors independent of CDK9 recruitment. Mol Cell 67:5–18. doi:10.1016/j.molcel.2017.06.004 28673542 PMC5663500

[77] Muhar M , Ebert A , Neumann T , Umkehrer C , Jude J , Wieshofer C , Rescheneder P , Lipp JJ , Herzog VA , Reichholf B , Cisneros DA , Hoffmann T , Schlapansky MF , Bhat P , von Haeseler A , Köcher T , Obenauf AC , Popow J , Ameres SL , Zuber J . 2018. SLAM-seq defines direct gene-regulatory functions of the BRD4-MYC axis. Science 360:800–805. doi:10.1126/science.aao2793 29622725 PMC6409205

[78] Ozer HG , El-Gamal D , Powell B , Hing ZA , Blachly JS , Harrington B , Mitchell S , Grieselhuber NR , Williams K , Lai T-H , Alinari L , Baiocchi RA , Brinton L , Baskin E , Cannon M , Beaver L , Goettl VM , Lucas DM , Woyach JA , Sampath D , Lehman AM , Yu L , Zhang J , Ma Y , Zhang Y , Spevak W , Shi S , Severson P , Shellooe R , Carias H , Tsang G , Dong K , Ewing T , Marimuthu A , Tantoy C , Walters J , Sanftner L , Rezaei H , Nespi M , Matusow B , Habets G , Ibrahim P , Zhang C , Mathé EA , Bollag G , Byrd JC , Lapalombella R . 2018. BRD4 profiling identifies critical chronic lymphocytic leukemia oncogenic circuits and reveals sensitivity to PLX51107 a novel structurally distinct BET inhibitor. Cancer Discov 8:458–477. doi:10.1158/2159-8290.CD-17-0902 29386193 PMC5882533

[79] Cribbs AP , Filippakopoulos P , Philpott M , Wells G , Penn H , Oerum H , Valge-Archer V , Feldmann M , Oppermann U . 2021. Dissecting the role of BET bromodomain proteins BRD2 and BRD4 in human NK cell function. Front Immunol 12:626255. doi:10.3389/fimmu.2021.626255 33717143 PMC7953504

[80] Evans EL , Becker JT , Fricke SL , Patel K , Sherer NM . 2018. HIV-1 Vif’s capacity to manipulate the cell cycle is species specific. J Virol 92:e02102-17. doi:10.1128/JVI.02102-17 29321323 PMC5972884

[81] Vansant G , Bruggemans A , Janssens J , Debyser Z . 2020. Block-and-lock strategies to cure HIV infection. Viruses 12:84. doi:10.3390/v12010084 31936859 PMC7019976

[82] Mousseau G , Clementz MA , Bakeman WN , Nagarsheth N , Cameron M , Shi J , Baran P , Fromentin R , Chomont N , Valente ST . 2012. An analog of the natural steroidal alkaloid cortistatin a potently suppresses tat-dependent HIV transcription. Cell Host Microbe 12:97–108. doi:10.1016/j.chom.2012.05.016 22817991 PMC3403716

[83] Mousseau G , Aneja R , Clementz MA , Mediouni S , Lima NS , Haregot A , Kessing CF , Jablonski JA , Thenin-Houssier S , Nagarsheth N , Trautmann L , Valente ST , Ott M , Prasad VR . 2019. Resistance to the tat inhibitor didehydro-cortistatin a is mediated by heightened basal HIV-1 transcription. mBio 10:e01750-18. doi:10.1128/mBio.01750-18 31266880 PMC6606815

[84] Luznik L , Kraus G , Guatelli J , Richman D , Wong-Staal F . 1995. Tat-independent replication of human immunodeficiency viruses. J Clin Invest 95:328–332. doi:10.1172/JCI117660 7814633 PMC295435

[85] Barboric M , Taube R , Nekrep N , Fujinaga K , Peterlin BM . 2000. Binding of tat to TAR and recruitment of positive transcription elongation factor b occur independently in bovine immunodeficiency virus. J Virol 74:6039–6044. doi:10.1128/JVI.74.13.6039-6044.2000 10846086 PMC112101

[86] Bogerd HP , Wiegand HL , Bieniasz PD , Cullen BR . 2000. Functional differences between human and bovine immunodeficiency virus tat transcription factors. J Virol 74:4666–4671. doi:10.1128/JVI.74.10.4666-4671.2000 10775603 PMC111987

[87] Oven I , Brdicková N , Kohoutek J , Vaupotic T , Narat M , Peterlin BM . 2007. AIRE recruits P-TEFb for transcriptional elongation of target genes in medullary thymic epithelial cells. Mol Cell Biol 27:8815–8823. doi:10.1128/MCB.01085-07 17938200 PMC2169392

[88] Shiozaki Y , Okamura K , Kohno S , Keenan AL , Williams K , Zhao X , Chick WS , Miyazaki-Anzai S , Miyazaki M . 2018. The CDK9–cyclin T1 complex mediates saturated fatty acid–induced vascular calcification by inducing expression of the transcription factor CHOP. J Biol Chem 293:17008–17020. doi:10.1074/jbc.RA118.004706 30209133 PMC6222109

[89] Becker JT , Sherer NM , Kirchhoff F . 2017. Subcellular localization of HIV-1 gag-pol mRNAs regulates sites of virion assembly. J Virol 91:e02315-16. doi:10.1128/JVI.02315-16 28053097 PMC5331792

[90] Müller B , Daecke J , Fackler OT , Dittmar MT , Zentgraf H , Kräusslich H-G . 2004. Construction and characterization of a fluorescently labeled infectious human immunodeficiency virus type 1 derivative. J Virol 78:10803–10813. doi:10.1128/JVI.78.19.10803-10813.2004 15367647 PMC516407

[91] Behrens RT , Aligeti M , Pocock GM , Higgins CA , Sherer NM , Kirchhoff F . 2017. Nuclear export signal masking regulates HIV-1 rev trafficking and viral RNA nuclear export. J Virol 91:e02107-16. doi:10.1128/JVI.02107-16 27852860 PMC5244332

[92] Fouchier RA , Meyer BE , Simon JH , Fischer U , Malim MH . 1997. HIV-1 infection of non-dividing cells: evidence that the amino-terminal basic region of the viral matrix protein is important for gag processing but not for post-entry nuclear import. EMBO J 16:4531–4539. doi:10.1093/emboj/16.15.4531 9303297 PMC1170079

[93] Gallois-Montbrun S , Kramer B , Swanson CM , Byers H , Lynham S , Ward M , Malim MH . 2007. Antiviral protein APOBEC3G localizes to ribonucleoprotein complexes found in P bodies and stress granules. J Virol 81:2165–2178. doi:10.1128/JVI.02287-06 17166910 PMC1865933

[94] O’Doherty U , Swiggard WJ , Malim MH . 2000. Human immunodeficiency virus type 1 spinoculation enhances infection through virus binding. J Virol 74:10074–10080. doi:10.1128/JVI.74.21.10074-10080.2000 11024136 PMC102046

[95] Gibellini D , Vitone F , Gori E , La Placa M , Re MC . 2004. Quantitative detection of human immunodeficiency virus type 1 (HIV-1) viral load by SYBR green real-time RT-PCR technique in HIV-1 seropositive patients. J Virol Methods 115:183–189. doi:10.1016/j.jviromet.2003.09.030 14667534

[96] Kim D , Paggi JM , Park C , Bennett C , Salzberg SL . 2019. Graph-based genome alignment and genotyping with HISAT2 and HISAT-genotype. Nat Biotechnol 37:907–915. doi:10.1038/s41587-019-0201-4 31375807 PMC7605509

[97] Anders S , Pyl PT , Huber W . 2015. HTSeq--a python framework to work with high-throughput sequencing data. Bioinforma Oxf Engl 31:166–169. doi:10.1093/bioinformatics/btu638 PMC4287950 25260700

[98] Love MI , Huber W , Anders S . 2014. Moderated estimation of fold change and dispersion for RNA-seq data with DESeq2. Genome Biol 15:550. doi:10.1186/s13059-014-0550-8 25516281 PMC4302049

